# Scalable one-pot synthesis of high-purity silver nanowires *via* controlled reaction kinetics for transparent conductive ink applications

**DOI:** 10.1039/d6ra03602c

**Published:** 2026-08-19

**Authors:** Umesh Pratap Pandey, Yashowanta N. Mohapatra, Ashish Gupta

**Affiliations:** a National Centre for Flexible Electronics, Indian Institute of Technology Kanpur Kanpur-208016 India ash@iitk.ac.in; b Department of Physics, Indian Institute of Technology Kanpur Kanpur-208016 India

## Abstract

Milligram-scale synthesis of nanomaterials needs re-optimization to be translated to gram-scale production. Increasing the reaction volume introduces additional kinetic variables that significantly influence product formation. By systematically addressing these parameters, a simplified protocol based on a modified one-pot polyol synthesis of AgNWs was developed. The method was successfully extended from an optimised small-scale reaction (0.11 g precursor loading/batch) to a 120-fold scale-up (13.2 g precursor loading/batch) without compromising morphology or yield. Notably, the process employs a cost-effective and low-purity precursor (AgNO_3_, 99.5% purity) instead of the ultra-high-purity precursor (99.9999%) used in the 0.11 g scale reaction while maintaining comparable product quality. The synthesis requires 40–150 min of reaction time depending on the batch size and consistently delivers high isolated yields of 91.0% ± 0.8% with excellent reproducibility. Two well-defined AgNW morphologies were obtained: short nanowires (10–15 µm length and 45 ± 10 nm diameter) and long nanowires (20–42 µm length and 80 ± 10 nm diameter). To demonstrate their practical applicability, the synthesised AgNWs were formulated into conductive inks compatible with non-porous PET substrates. Uniform wire-bar-coated transparent conductive films exhibited an optical transmittance of 88.4% w.r.t PET and an average sheet resistance of 16.23 Ω sq^−1^, confirming their suitability for applications in solution-processed transparent electrodes and flexible electronic devices.

## Abstract 2

Milligram-scale synthesis of nanomaterials needs re-optimization to be translated to gram-scale production.

## Graphical abstract

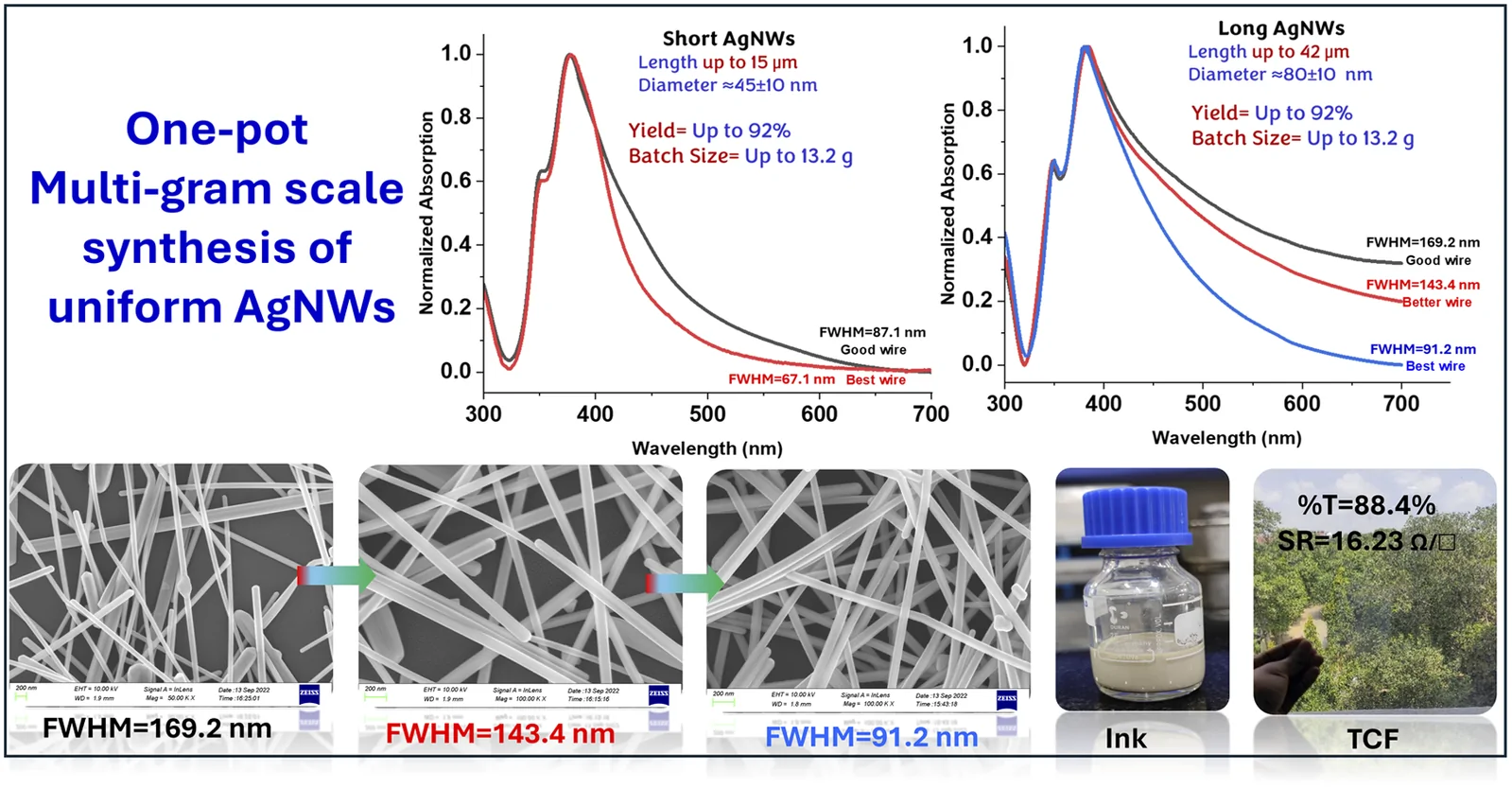

## 1 Introduction

Silver nanowires (AgNWs) have been widely explored in flexible electronics for coating and printing transparent conductive electrodes (TCEs) and devices due to their high optical transmittance, low sheet resistance and compatibility with low-temperature solution-processing.^1–4^ AgNW networks can be fabricated from AgNW-based inks through scalable techniques such as bar coating, spray deposition, and printing.^5^ However, achieving uniform functionality in each batch of the ink requires the synthesis of large batches of AgNWs with same morphology and electrical properties.^6,7^ Most laboratory-scale nanomaterial synthesis strategies are typically optimized at the milligram to gram scale,^8,9*a*,*b*,10*a*–*f*^ whereas industrial processes are designed for large-scale production using dedicated, highly engineered systems.^11,12^ Bridging the gap between laboratory-scale and industrial scale strategies remains a significant challenge in nanochemistry. In a typical small R&D laboratory setting, however, there is a practical need for an intermediate production scale, *i.e.*, in a 5–20 gram^13^ batch size. Despite its importance, the intermediate scale is relatively underexplored in the literature, creating a critical gap between laboratory research and industrial implementation.

Increasing the reaction volume from the milligram to gram scale introduces additional kinetic variables that significantly influence both morphology and yield.^14–19^ However, with different strategies put forward to control morphology, size distribution, and overall yield, “large-scale synthesis” can be achieved.

True industrial-scale production demonstrated by Kulkarni and co-workers approached industrial relevance through engineered process control and yielded ≈500 g per day and above.^11,12^ In contrast, there are many studies that, despite being conducted at the laboratory scale (mg to <2 g), described their methods as “large-scale”, including studies by Zeng and Zhang (0.9 g AgNO_3_ per 100 ml of ethylene glycol (EG)), Seung Hwan Ko (15 ml of 0.094 M AgNO_3_), and Qiao (25 ml autoclave reactions).^20–22^ While such claims may be valid within a laboratory context, they remain small-scale from an R&D perspective. A multigram synthesis exemplified by Zhang and co-workers, using 30 g of AgNO_3_ in 3.6 L of EG with staged temperature control and NiCl_2_ seeding, represents a crucial bridge between laboratory and industrial production.^23^ They performed the reaction at two different temperatures. The seeds were prepared at 100 °C, and the growth of AgNWs was achieved at 120 °C–180 °C. While these strategies demonstrate the feasibility of increasing the batch size, they involve additional operational complexity, stringent process control, or multistep purification. Therefore, there remains a need for a robust and scalable protocol based on controlled reaction kinetics to enable the synthesis of impurity-free silver NWs.^25,26^

The synthesis of AgNWs is described as a two-step process: (i) the formation of multiple twinned silver seeds and (ii) the anisotropic growth of these seeds into one-dimensional NWs.^24^ We anticipated that controlling the non-uniform nucleation and growth could minimize batch-to-batch difference and polydispersity. During scale-up, the control of nucleation becomes particularly challenging.^17,18^ In small reaction volumes, reagents are rapidly mixed and supersaturation develops almost uniformly, often enabling the burst nucleation mechanism that produces monodisperse nanoparticles. In contrast, a larger reaction vessel tends to generate spatially non-uniform supersaturation zones due to slower mixing and delayed reagent distribution. As a result, nucleation may occur at different times and locations within the reactor, following a complex mechanism and leading to broader particle size distributions and secondary nucleation events.^25^

**Fig. 1:**
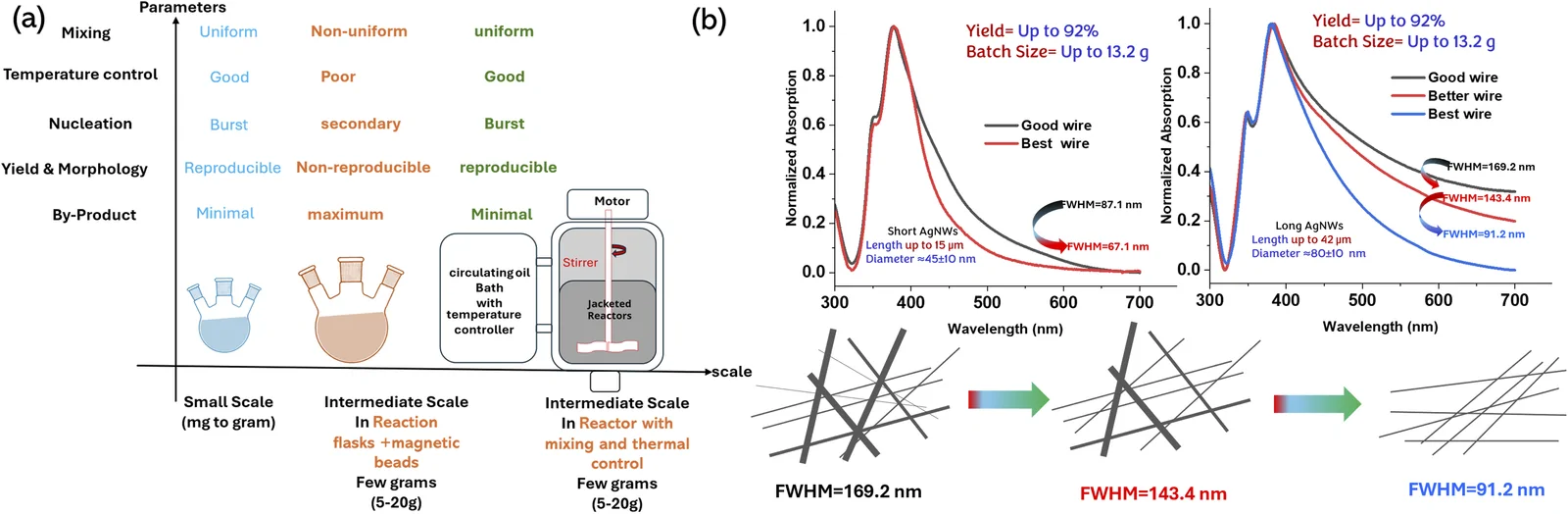
(a) Factors affecting the scale of the reaction. (b) Summary of the optimization of the present work. Two types of NWs, short AgNWs (S-AgNWs) and long AgNWs (L-AgNWs), were synthesized with >92% yield and up to 13.2 g scale with the least amount of by-products. The full width at half maxima (FWHM) shows the homogeneous size with the least amount of by-products.

Nucleation and growth kinetics^17,26,27^ are strongly influenced by the reaction scale (Fig. 1a). After nucleation, particle growth proceeds *via* diffusion-controlled processes that highly depend on concentration gradients, temperature distribution and mass transport. In milligram-scale reactions, these factors are usually well regulated due to short diffusion lengths and efficient mixing. However, at the gram scale, diffusion limitations and concentration gradients become significant, altering growth pathways and influencing key morphological parameters such as nanowire diameter, aspect ratio and crystal anisotropy.

Mass transfer and heat transfer limitations further complicate the scale-up process. In small-scale reactions, heat is typically dissipated efficiently due to the high surface area-to-volume ratio of the reaction vessel. In contrast, larger reaction volumes often generate temperature gradients and localized hot spots. Such thermal inhomogeneities may shift equilibrium conditions and alter reaction kinetics, leading to non-uniform nucleation and growth behaviour. Likewise, mixing time increases significantly with reactor volume, often disrupting the burst nucleation regime that is essential for controlled nanomaterial formation. As a result, scale-up frequently results in continuous nucleation, aggregation and polydispersed products.

We anticipated that batch upscaling could be achieved by controlling the reaction kinetics, particularly by uniform mixing and tuning concentration and temperature gradients according to the reaction mechanism. We upscaled a method that (i) originates from a well-optimized small-scale protocol, (ii) minimizes by-product formation, (iii) employs a simple and reproducible reaction setup, (iv) reduces processing time, and (v) limits the number of critical control variables during scale-up.

We drew insights from the report by Baldi *et al.*,^28^ who employed a modified polyol method for the synthesis of AgNWs and demonstrated a one-pot synthesis at a 0.11 g scale, achieving minimal by-product formation and improved purity compared with the earlier reported methods. Interestingly, we utilized comparatively less pure starting materials and achieved comparable results at 120 times larger reaction (13.2 g) scale by optimizing the reaction parameters, particularly temperature, and mixing parameters (Fig. 1a and b). Highly pure AgNO_3_ (99.9999% pure) was employed in most of the earlier works.^28,29^ In the present work, we employed 99.5% pure AgNO_3_ without compromising the purity of the product. Furthermore, we extended the idea by synthesising nanowires with thicker and longer morphologies. While small-scale synthesis (up to the gram scale) was achieved without significant challenges due to effective control over stirring and temperature, scaling to an intermediate range (up to ∼4.4 g scales) introduced difficulties in obtaining consistent product quality. To address these issues, synthesis was transitioned to a 3 L reactor, enabling the processing of up to 13.2 g of precursor after optimization. The use of the reactor provided improved temperature control and more efficient mixing, resulting in enhanced reproducibility and uniform product characteristics in all the batches (Fig. 1a and S6).

Herein, we demonstrate a controlled scale-up of the polyol synthesis, along with the systematic characterization and post-synthetic processing of two distinct AgNW morphologies. The process was successfully optimized over a precursor loading range from 0.11 g to 13.2 g while preserving the nanowires' morphological uniformity, aspect ratio, and electrical functionality, achieving isolated yields of 91–92%. The first type of NWs exhibited lengths in the range of 10–15 µm and diameters of 45 ± 10 nm, whereas the second type of nanowires exhibited lengths between 20 and 42 µm and diameters of 80 ± 10 nm (Fig. S1, S2 and S4). The synthesized AgNWs were subsequently formulated into conductive inks compatible with polyethylene terephthalate (PET). The transparent conductive film was printed with 88.4% optical transparency w.r.t PET, and the average sheet resistance was 16.23 Ω sq^−1^.

## 2 Experimental section

### 2.1 Materials

All reagents were used as received without further purification. Sodium chloride (NaCl, >99%), silver nitrate (AgNO_3_, >99.5%), and ethylene glycol (EG, 99.8%) were obtained from Merck. Poly(vinylpyrrolidone) (PVP, *M*_w_ ≈ 40 kDa, 98%) was purchased from Sisco Reseach Laboratories Pvt. Ltd (SRL), India and high-molecular-weight PVP (*M*_w_ ≈ 1300 kDa, 99%) was obtained from Sigma-Aldrich. Ethylene glycol (EG) and AgNO_3_ were stored under ambient laboratory conditions. Deionized (DI) water was produced in-house and used throughout all experiments.

### 2.2 Synthesis of silver chloride (AgCl)

AgCl was prepared immediately prior to use to ensure the reproducibility of the NW morphology. Owing to its photosensitivity, all steps, including precipitation, washing, and drying, were performed under dark conditions.

An aqueous solution of AgNO_3_ was mixed with an aqueous NaCl solution in a 1 : 2 molar ratio under stirring for 2 min, resulting in the rapid formation of a white precipitate of AgCl. The solid was separated from the supernatant, washed twice with DI water to remove residual ions, and dried under vacuum in the dark. The freshly prepared AgCl was stored in an amber bottle wrapped with aluminium foil in a dry environment. Only freshly prepared AgCl was used for each synthesis as ageing of the salt was found to adversely affect the NW morphological uniformity.

### 2.3 Synthesis of AgNWs

The polyol method was employed with systematic scale variations. Short-AgNWs (S-AgNWs) were first optimized at the 0.11 g precursor scale and subsequently scaled by factors of 10× (1.1 g), 20× (2.2 g), 40× (4.4 g), 80× (8.8 g), and 120× (13.2 g). After establishing the optimized conditions for S-AgNWs, long AgNWs (L-AgNWs) were synthesized at the 8.8 g and 13.2 g scales. The weights of AgNO_3_, AgCl, PVP and EG used at different reaction scales are given in Table 1. For reaction scales up to 2.2 g, syntheses were conducted in a three-neck round-bottom (RB) flask immersed in an oil bath with magnetic stirring. However, at ≥4.4 g scales, the reaction mixture became highly viscous after the addition of PVP and AgNO_3_, leading to inefficient mixing and heat transfer in the RB setup. Therefore, reactions at the 4.4 g scale and above were performed in a 3 L double-walled glass reactor equipped with an integrated setup for external heating and overhead mechanical stirring.

**Table 1:** Amounts of silver nitrate, silver chloride, PVP and EG at different reaction scales, along with time, FWHM and reaction parameters. For small Ag wires (S-AgNWs), PVP *M*_w_ = 40 kDa and for large Ag nanowires (L-AgNWs), PVP *M*_w_ = 1300 kDa. The average yield of the reproducibility batch of L-AgNWs was found to be 91.0% ± 0.8%

Type	Remark	AgNO_3_	AgCl	FWHM range	Isolated yield (±0.5%)	Vessel + stirring method	Time (min)	PVP (g)	EG (mL)	AgCl (mg)	Figure ref.
S-AgNWs	AgCl effect	0.11 g	Stored AgCl	174.1	—	RB flask + magnetic	40	0.34	25	25	Fig. 3A
	Scaling	0.11 g	Fresh AgCl	87.1	92%	RB flask + magnetic	40	0.34	25	25	Fig. 3A
	Scaling	1.1 g	Fresh AgCl	74.4	92%	RB flask + magnetic	40	3.40	200	250	Fig. 3D
	Scaling	2.2 g	Fresh AgCl	73.8	91%	RB flask + magnetic	40		400	500	Fig. 3D
	Vessel shape	4.4 g	Fresh AgCl	178.1	—	RB flask + magnetic	65	13.5	800	1000	Fig. 3B
	Scaling	4.4 g	Fresh AgCl	78.0	92%	3 L reactor + mechanical	65	13.5	800	1000	Fig. 3B
	Scaling	8.8 g	Fresh AgCl B1	67.1	92%	3 L reactor + mechanical	65	27.0	1600	2000	Fig. 3D
	Reproducibility	8.8 g	Fresh AgCl B2	69.0	91%	3 L reactor + mechanical	65	27.0	1600	2000	Fig. 3D
	Scaling	13.2 g	Fresh AgCl B1	71.5	91%	3 L reactor + mechanical	65	40.0	2400	3000	Fig. 3D
	Reaction time	8.8 g	Fresh AgCl		91%	3 L reactor + mechanical	65	27.0	1600	2000	Fig. 3C
				70.0	—		5th min				
				119.2	—		35th min				
				86.1	—		65th min				
L-AgNWs	AgCl effect	8.8 g	Fresh AgCl	169.2	86%	3 L reactor + mechanical	150	27.0	2000	2000	Fig. 4A
	Scaling	8.8 g	Stored AgCl	213.6	—	3 L reactor + mechanical	150	27.0	2000	2000	
	Reaction time	8.8 g	Fresh AgCl		—	3 L reactor + mechanical	150	27.0	2000	2000	Fig. 4B
				140.4	—		60th min				
				137.5	—		90th min				
				122.7	—		120th min				
				118.5	—		150th min				
	Optimization	13.2 g	Fresh AgCl	159.6	88%	3 L reactor + mechanical	150	40.0	2600	3000	Fig. 4C
	Optimization	13.2 g	Fresh AgCl	169.2	87%	3 L reactor + mechanical	150	40.0	2600	3000	Fig. 4C
	Optimization	8.8 g	Fresh AgCl	152.2	88%	3 L reactor + mechanical	150	27.0	2000	2000	Fig. 4C
	Optimization	8.8 g	Fresh AgCl	145.9	89%	3 L reactor + mechanical	150	27.0	2000	2000	Fig. 4C
	Optimization	8.8 g	Fresh AgCl	147.4	88%	3 L reactor + mechanical	150	27.0	2000	2000	Fig. 4C
	Optimization	8.8 g	Fresh AgCl	143.4	89%	3 L reactor + mechanical	150	27.0	2000	2000	Fig. 4C
	Reproducibility	8.8 g	Fresh AgCl B1	91.2	92%	3 L reactor + mechanical	150	27.0	2000	2000	Fig. 4D
	Reproducibility	8.8 g	Fresh AgCl B2	91.6	92%	3 L reactor + mechanical	150	27.0	2000	2000	Fig. 4D
	Reproducibility	8.8 g	Fresh AgCl B3	97.4	91%	3 L reactor + mechanical	150	27.0	2000	2000	Fig. 4D
	Reproducibility	8.8 g	Fresh AgCl B4	101.6	90%	3 L reactor + mechanical	150	27.0	2000	2000	Fig. 4D
	Reproducibility	13.2 g	Fresh AgCl B1	102.9	91%	3 L reactor + mechanical	150	27.0	2000	2000	Fig. 4D
	Reproducibility	13.2 g	Fresh AgCl B2	107.3	91%	3 L reactor + mechanical	150	27.0	2000	2000	Fig. 4D
	Reproducibility	13.2 g	Fresh AgCl B3	107.0	90%	3 L reactor + mechanical	150	27.0	2000	2000	Fig. 4D

In a representative synthesis (≤2.2 g scales), the required amount of PVP (Table 1) was dissolved in EG in a three-neck RB flask and heated to 160 °C under a continuous N_2_ flow with stirring at 800 rpm. Upon reaching thermal equilibrium, freshly ground AgCl was added in a single portion. Within ∼3 min, the solution changed from colourless to pale yellow, indicating initial nucleation. Subsequently, AgNO_3_ was introduced rapidly in one portion, and the flask was sealed with septa or ground-glass stoppers and secured with parafilm. The reaction temperature was maintained at 160 °C for 40 min (2.2 g scale synthesis of S-AgNWs) under constant stirring. Reaction progress was monitored by withdrawing aliquots. The number of reactants, PVP, time and solvent at different reaction scales, along with the full width at half maximum (FWHM) and reaction parameters are given in Table 1. For ≥4.4 g scales, the same chemical sequence was followed in the glass reactor. The influence of the parameters such as mixing protocol, N_2_ flow rate, and temperature fluctuations on the NW morphology is discussed in the results and discussion section.

### 2.4 Isolation, purification and storage of AgNWs

After the completion of the reaction, the mixture was cooled to 25 °C and allowed to stand for 1 h to enable the gravitational settling of excess AgCl aggregates. AgNWs were isolated *via* solvent-mediated selective precipitation by the gradual addition of acetone under continuous stirring. Owing to the poor solubility of PVP in acetone (<0.1 g mL^−1^ at 25 °C), solvent exchange induced the collapse of the PVP solvation shell, resulting in the rapid agglomeration and precipitation of PVP-coated NWs, while smaller nanoparticles remained suspended. The precipitate was collected and washed twice with acetone (1 : 5 v/v) to remove any residual nanoparticles and unreacted species. The SEM analysis of the unwashed samples revealed a thick PVP shell (∼15–20 nm). Thermogravimetric analysis (TGA) indicated 18.1 wt% residual PVP in the NWs after initial precipitation, corresponding to increased apparent diameters (110–125 nm). Successive washing for 3 cycles followed by centrifugation (3000 rpm, 15 min) reduced the PVP content to 4.3 wt% in the nanowires, and further washing reduced the PVP content to 0.01 wt%. However, 0.01 wt% PVP ethanolic solution precipitated within a few minutes (Fig. 7B). Excessive sonication was avoided as mechanical fragmentation of the NWs was observed. While polymer removal enhances conductivity, complete stripping compromises colloidal stability and accelerates oxidative degradation.

The storage stability was systematically evaluated. Drying led to irreversible agglomeration and wire breakage upon redispersion. Wet storage in 1 wt% ethyl cellulose (EC)/EtOH delayed but did not prevent precipitation and fragmentation.

Optimal stability was achieved by storing AgNWs in EG containing excess PVP, with suppressed oxidation and aggregation. Under these conditions, the NWs remained stable for at least five months. During washing and centrifugation, small nanowires eliminated, and this resulted in a decrease in FWHM (105 nm to 97 nm) (Fig. S3). Storing AgNWs in the EG-PVP mixture confirms the structural preservation of the NWs.

### 2.5 Yield calculation

The yield of AgNWs was defined as the percentage of silver atoms from AgNO_3_ converted into the NWs, excluding the nanoparticles and unreacted precursors. Detailed calculations are provided in the SI.

The formulae are as follows:Yield (%) = (Experimental yield of AgNWs)/(0.6350 × mass of AgNO_3_) × 100.Experimental yield = (weight of AgNWs) − (weight of PVP calculated by TGA).

0.6350 is the mass fraction of the elemental silver in AgNO_3_. M(AgNO_3_)/M(Ag) = 169.87/107.87 = 0.6350.

Although AgCl is added to the reaction mixture, its primary role is to regulate nucleation through controlled dissolution and seed formation. Only a very small quantity of AgCl is employed compared with that of AgNO_3_, and its contribution to the final nanowire mass is very less relative to the total silver supplied by AgNO_3_.

### 2.6 Preparation of the AgNW conductive ink

#### 2.6.1 Preparation of the EC stock solution (10 wt%)

A 10 wt% EC solution was prepared in a toluene–ethanol solvent mixture (7 : 3 w/w). Briefly, 90 g of the solvent mixture was taken in a beaker, and 10 g of EC was gradually added under continuous stirring to avoid agglomeration. The solution was stirred at room temperature overnight until a clear and homogeneous stock solution was obtained.

#### 2.6.2 Formulation of the AgNW ink

A 100 g batch of the AgNW ink was prepared by combining 60.0 g of an AgNW dispersion (5 wt% in EtOH) with 15.0 g of 10 wt% EC stock solution. Subsequently, 25.0 g of EtOH was added under continuous stirring to achieve the desired composition and prevent NW aggregation. The mixture was stirred for 40 min and then sonicated for 10 min in a water bath to obtain a homogeneous and stable conductive ink suitable for coating and printing applications.

### 2.7 Characterization

The optical properties of AgNWs and the transparency of the conductive films were measured using a UV-vis spectrophotometer (Lambda 35, PerkinElmer) in the wavelength range of 280–700 nm. Morphological analysis was performed using field-emission scanning electron microscopy (FESEM, Zeiss SUPRA 40 VP) and high resolution transmission electron microscopy (HRTEM, Titan G2 60-300 kV). PXRD was performed using a Rigaku MiniFlex 600 benchtop X-ray diffractometer equipped with a sealed copper (Cu) anode tube as the X-ray source (*λ* ∼ 1.5418 Å). The sheet resistance of the AgNW films was measured using a four-probe system (Ossila 2018). Centrifugation was carried out using a Hermle Z36HK refrigerated centrifuge.

UV-vis spectroscopy was used to monitor the structural evolution of AgNWs by evaluating the SPR bands. The position, intensity, and bandwidth of the SPR bands depend on the particle size and shape, enabling the rapid assessment of morphology and anisotropy during synthesis. For quasi-spherical Ag nanoparticles, a single SPR band appears at ∼415–420 nm. In anisotropic NWs, this band splits into transverse (∼350 nm) and longitudinal (∼380 nm or higher) modes. The longitudinal mode is red-shifted and strongly dependent on the NW aspect ratio and length, reflecting anisotropic plasmonic behaviour. Consequently, the systematic monitoring of the SPR band positions and their FWHM enables the qualitative and semi-quantitative assessment of NW growth, dimensional uniformity and by-product formation. Thus, UV-vis spectroscopy serves as a convenient tool to track the seed-to-nanowire transformation and evaluate the morphological purity during AgNW synthesis.

## 3 Results and discussion

**Fig. 2:**
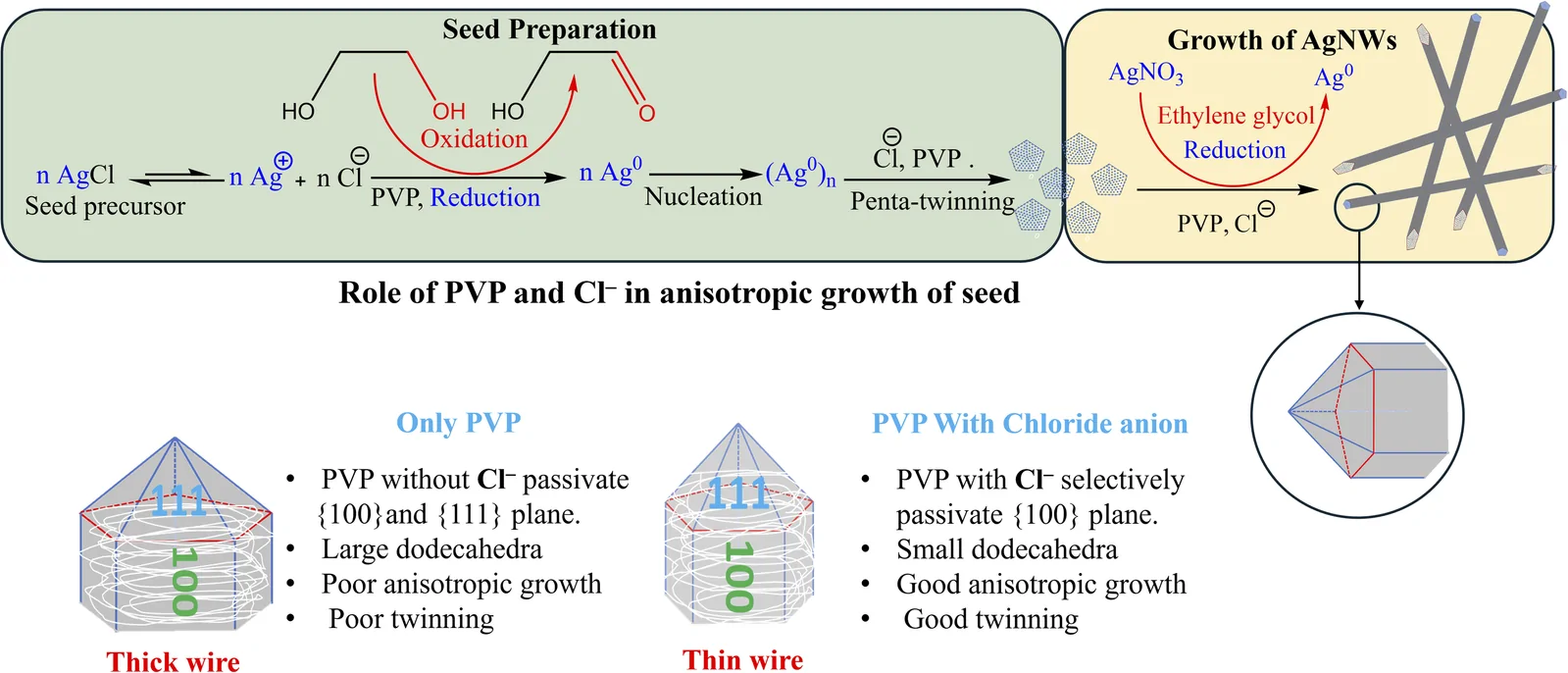
Schematic of the mechanism of AgNW synthesis and the role of PVP and chloride ions in capping and twinning. The first step is to prepare the seed (1), and the 2nd step is the growth of the NWs (2). PVP alone passivates the 111 and 100 planes. However, in the presence of chloride ions, it is highly selective for the 100 planes. This selectivity results in the formation of thin nanowires.

The formation of by-products during the scale-up of the AgNW synthesis from the milligram to gram scale can be minimized by the precise control of the critical reaction variables. The underlying reaction mechanism of the AgCl-assisted AgNW synthesis *via* the polyol method can be rationalized by correlating the experimental observations with the earlier reports in the literature (Fig. 2).^17,26,27^ The synthesis is generally described as a two-step process: (i) the formation of multiply twinned silver seeds and (ii) the anisotropic growth of these seeds into one-dimensional NWs.

Seed purity and dissolution kinetics play decisive roles in determining the nucleation uniformity, growth selectivity, and spectral homogeneity of AgNWs. Variations in the physicochemical state of AgCl directly influence the initial reduction dynamics and, consequently, the morphology of the final product. Multiple strategies have been reported for generating pentagonal twinned seeds, including microfluidic droplet-based AgCl seeding,^30^ transition-metal-assisted twinning, and halide-mediated approaches.^31^ Microfluidic methods enable the precise modulation of AgNO_3_ and NaCl concentrations to tailor the AgCl seed size and, consequently, AgNW dimensions; however, these approaches require specialised instrumentation and involve complex handling procedures. Transition-metal-mediated routes (for example, those mediated by Pt, Fe, Ni, and Co salts) promote anisotropic twinning but introduce additional chemical variables^32,33^ In this study, AgCl was selected as an *in situ* seed precursor owing to its simplicity, cost-effectiveness, and minimal reaction variables.

Choosing one-pot synthesis provides extra advantages and better controls the critical variables. Seed formation typically occurs at a relatively lower temperature, while subsequent NW elongation proceeds at higher temperatures under conditions favouring kinetically controlled, facet-selective growth.^13^ The reducing strength of EG increases with temperature due to enhanced oxidation to glycolaldehyde, accelerating Ag^+^ reduction and wire elongation. In most reported protocols, these two stages are carried out in a two-pot configuration to allow independent control over the nucleation and growth parameters. However, such approaches increase the number of critical variables (*e.g.*, precursor concentration, temperature ramping, transfer losses, and aging time), which can complicate reproducibility during scale-up. To minimize process complexity and improve scalability, both seed generation and nanowire growth were performed in a one-pot configuration at a constant reaction temperature. This strategy reduces the number of handling steps, limits batch-to-batch variability and provides better suitability for gram-scale synthesis.

Fresh AgCl is readily synthesised, by mixing AgNO_3_ with NaCl in the dark, within few seconds.^28^ Furthermore, during seed formation, it does not require an additional vessel or specific temperature. In EG, AgCl slowly dissociates to release the Ag^+^ and Cl^−^ ions, thereby maintaining a low and controlled Ag^+^ concentration in the solution even at a higher reaction scale. This gradual ion release regulates the reduction of Ag^+^ to Ag^0^, which is essential for producing uniform seeds. The slow dissociation of AgCl allows the Ag^+^ concentration to increase progressively until it reaches the critical supersaturation level. Once this threshold is exceeded, a burst nucleation event occurs, leading to the rapid formation of nuclei. Because the ion release is gradual and controlled, nucleation occurs within a narrow size window, promoting uniform seed formation and reducing secondary nucleation. Within 3 minutes of reaction, Ag^0^ seed formation completes.

AgNO_3_ is added to the reaction mixture to provide a continuous source of Ag^+^ to be reduced and deposited for nanowire growth. The primary role of AgCl sis to regulate nucleation. Only a relatively small quantity of AgCl is employed compared with AgNO_3_, and its contribution to the final NW mass is very less relative to the total silver supplied by AgNO_3_. Hence, the yield is calculated based on only the AgNO_3_ amount. Under the mentioned reaction conditions, EG functions both as a solvent and a reducing agent. At elevated temperatures, EG undergoes thermal oxidation to glycolaldehyde (HO-CH_2_CHO), which acts as a strong reductant, and is further oxidised to glyoxal (OCHCHO). This redox sequence provides a continuous supply of Ag^0^ atoms. The controlled reduction of Ag^+^ to Ag^0^ is essential. Growth kinetics are markedly temperature-dependent.^34–36^ Comparable wire lengths were achieved at 160 °C but required substantially longer reaction times when the synthesis was carried out at 140 °C. While lower temperatures may yield thicker and longer wires under extended reaction times, higher temperatures above ∼160 °C not only promote faster growth but also induce heterogeneous nucleation and formation of undesirable nanoparticles. Baldi *et al.* observed a gradual sphere-to-rod transition near 160 °C, followed by a rapid rod-to-wire transformation.^28^ Based on these insights, 160 °C was selected as an optimal compromise to balance nucleation control and growth kinetics while minimizing secondary particle formation.

The role of Cl^−^ ions is multifaceted. Beyond regulating Ag^+^ availability by altering AgCl dissolution equilibria, halide ions are widely recognised to promote anisotropic growth and restrict lateral growth.^26^ It is well established that the molecular weight of PVP strongly influences the NW morphology. Lower molecular weight PVP (40 kDa, ≥99%) favours the formation of shorter and relatively thinner nanowires (S-AgNWs), while higher molecular weight PVP (1300 kDa, ≥99%) promotes extended axial growth, resulting in longer NWs (L-AgNWs).^37^

**Fig. 3:**
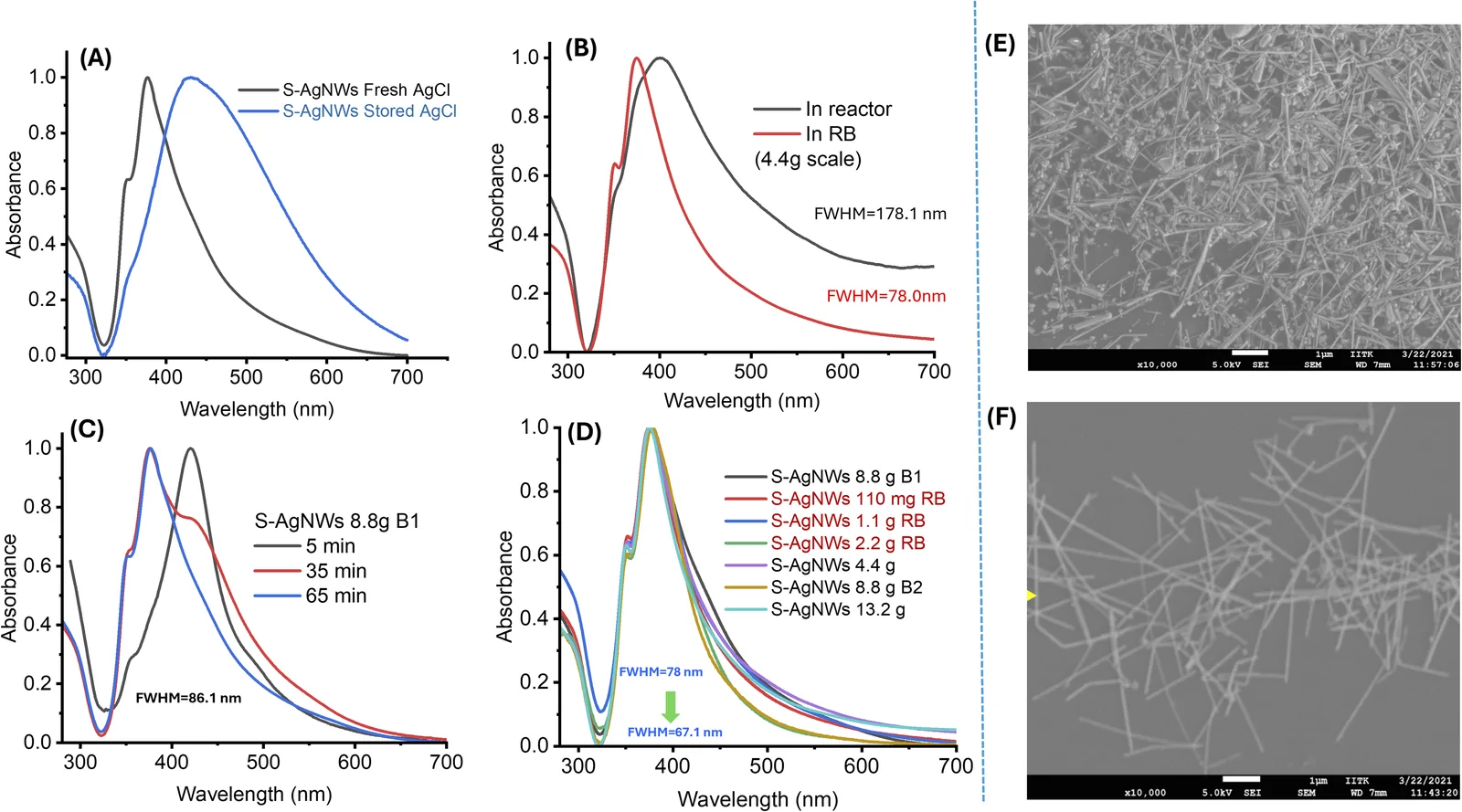
(A) UV-vis absorption spectra of AgNWs synthesized using fresh and stored AgCl. (B) Comparison of the AgNW synthesis performed in a round-bottom flask (RB) and a glass reactor at the 4.4 g scale, illustrating the effect of the reaction vessel. (C) Evolution of AgNWs at different growth times during synthesis at the 8.8 g scale. (D) AgNWs synthesized over reaction scales ranging from 0.11 to 13.2 g; reactions at ≤2.2 g scales were carried out in a round-bottom flask (RB), while those at ≥4.4 g scales were performed in a glass reactor. B1 and B2 denote the two independent batches synthesized under identical reaction conditions. (E) SEM image of AgNWs synthesized using stored AgCl. (F) SEM image of AgNWs synthesized using fresh AgCl (additional details are provided in Table 1).

Two types of AgNWs were synthesized by varying the molecular weight (*M*_w_) of PVP, solvent and reaction timing. Initially, we reproduced the synthesis procedure reported by Baldi *et al.* AgNWs were synthesised using stored and fresh AgCl. Prolonged storage of AgCl is expected to lead to partial photochemical degradation and the formation of metallic or oxidised Ag species, promoting uncontrolled secondary nucleation and resulting in inadequate growth. A broadened 174.1 nm FWHM of the UV-vis absorption profile (Fig. 3A) is observed, indicating a wider size distribution and increased by-product formation. This observation is consistent with the SEM image of the product (Fig. 3E).

Subsequently, freshly prepared AgCl was employed and the FWHM reduced drastically to 87.1 nm (Fig. 3A). We found similar yields and morphology upon increasing the scale 20×, from 0.11 g (yield 92%, FWHM 74.4 nm) to 2.2 g (yield 91%, FWHM 73.8 nm) (Fig. 3D, red notation). All small-scale syntheses (≤2.2 g of AgNO_3_) were carried out in a three-neck round-bottom flask equipped with a magnetic stirrer and immersed in an oil bath to ensure uniform heating. This configuration provided adequate thermal control and mixing efficiency at low reaction volumes. However, upon increasing the reaction scale to 4.4 g, non-uniform stirring was observed, which affected the nucleation uniformity and resulted in a broad UV-vis absorption FWHM (178.1 nm) with no significant yield (Fig. 3B).

To address these limitations, reactions at the 4.4 g and higher scales were conducted in a 3 L double-walled glass reactor equipped with an integrated heating system and overhead mechanical stirring. The double-layered design enabled efficient thermal regulation, while mechanical agitation ensured homogeneous mixing under high material loading. This modification provided improved temperature uniformity, enhanced mass transfer, and consistent growth kinetics during the scale-up process. For the products obtained under the abovementioned reaction conditions, the FWHM decreased to 78 nm (for the 4.4 g scale reaction in the 3 L reactor) from 178.1 nm (for the 4 g scale reaction in the RB), with an excellent yield of 92%. Interestingly, the 8.8 g scale reaction conducted in the aforementioned setup exhibited comparable results (yield and purity) to those reported by Baldi *et al.* at the 0.11 g scale (Fig. 3D and S6).

Furthermore, when AgCl is used with adequate grinding for uniform dispersion, the non-aggregated AgCl particles exhibited better dissolution kinetics in EG. Moreover, the strict regulation of the reaction temperature (160 °C) and maintenance of an inert N_2_ atmosphere minimised the oxidative side reactions and heterogeneous nucleation events. The uniform release of Ag^+^ caused homogeneous nucleation and regular growth, as reflected by the shortening of the FWHM (from 78 to 67.1 nm) in the absorption spectrum (Fig. 3D). This product has no additional morphology changes as clearly visible in the SEM image (Fig. 3F). These observations confirm that both the purity and grain size of AgCl are critical parameters. The freshly synthesised and finely dispersed AgCl ensured rapid and homogeneous dissolution in the initial stage of the reaction. This facilitated uniform pentagonal seed formation and controlled anisotropic growth, yielding AgNWs with a narrower FWHM and reduced secondary particle formation. Under these optimised conditions, reproducible growth kinetics and improved morphological uniformity were achieved (Fig. 3).

During the reaction, a characteristic colour change is observed: the initial turbid white suspension gradually turns yellow, corresponding to pentagonal seed formation;^28^ upon AgNO_3_ addition, the solution becomes light grey and eventually turns dark grey, consistent with progressive NW growth. The abovementioned change is directly related to the growth time, which is a critical kinetic parameter in the polyol-mediated synthesis of AgNWs as it directly governs the anisotropic elongation and ultimately determines the length and dispersity of NWs.

**Fig. 4:**
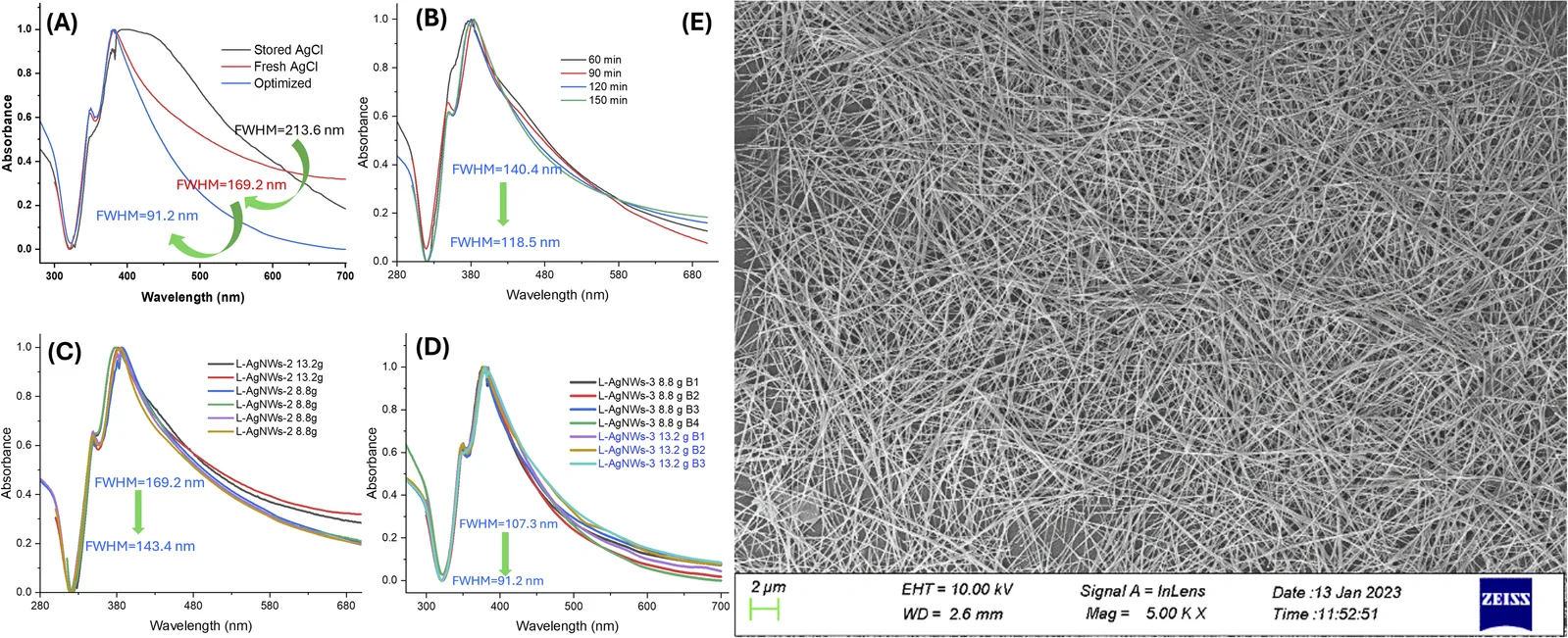
(A) UV-vis absorption spectra of the as-synthesized L-AgNWs prepared using stored AgCl and fresh AgCl and under the optimized synthesis conditions employing fresh AgCl. (B) Evolution of the time-dependent UV-vis absorption spectra of L-AgNWs during growth at the 8.8 g scale reaction. (C) UV-vis absorption spectra of the optimization batches of L-AgNWs synthesised at the 8.8 g and 13.2 g scales (D) Reproducibility evaluation using the UV-vis absorption spectra of the optimization batches of L-AgNWs synthesised at the 8.8 g and 13.2 g scales. B1, B2, and B3 denote three independent batches synthesized under identical reaction conditions. (E) Representative SEM image of the optimized L-AgNWs obtained under the best reaction conditions (additional details are provided in Table 1).

To elucidate its influence, the temporal evolution of the reaction was systematically monitored by correlating the reaction time with the FWHM of the characteristic surface plasmon resonance (SPR) bands in the UV-vis absorption spectra. The growth kinetics were investigated for both S-AgNWs (Fig. 3C) and L-AgNWs (Fig. 4B).

For S-AgNWs, aliquots were withdrawn at 5, 35, and 65 min during the reaction (Fig. 3C). At 5 min, the absorption spectrum exhibited a sharp and intense peak centred at ∼420 nm, accompanied by a weak shoulder at ∼350 nm. The dominant ∼420 nm band corresponds to the dipolar SPR of the quasi-spherical Ag nanoparticles, indicating that nucleation and early-stage isotropic growth predominated at this stage. After 35 min, the intensity of the ∼420 nm peak markedly decreased, while a new band emerged at ∼375 nm with a distinct shoulder at ∼350 nm. The appearance of these features is characteristic of AgNWs and arises from transverse plasmon modes associated with anisotropic nanostructures. This spectral transition confirms the onset of one-dimensional growth through selective facet stabilization.

Upon extending the reaction time to 65 min, the ∼420 nm band completely disappeared, and only the AgNW signature peaks at ∼375 nm with a shoulder near ∼350 nm persisted (Fig. 3C). Concomitantly, the FWHM narrowed, indicating reduced polydispersity and improved morphological uniformity. These observations suggest that 65 min is sufficient to achieve complete NW growth with minimal residual nanoparticles and limited by-product formation.

The optimized protocol was subsequently scaled up to a 13.2 g scale reaction for S-AgNW synthesis. The scaled-up reaction yielded comparable spectral features and morphology, demonstrating excellent reproducibility. The process was independently repeated at 8.8 g under identical conditions, producing consistent results and thereby confirming the robustness and scalability of the synthetic strategy (Fig. 3D).

### 3.1 Optimization of the synthesis of L-AgNWs

L-AgNWs were synthesised only at the 8.8 g and 13.2 g scales. To tailor the longer nanowire, three significant changes were made. First, the chain length of high-*M*_w_ PVP (1300 kDa, ≥99%) was used, facilitating stronger steric stabilization, more effective facet passivation, suppressed radial growth and higher aspect ratios.^37^ Second, the reaction medium was diluted to obtain enough volume for overcoming the lack of free space in the solution. Third, the growth time was increased to give enough duration for nanowire growth. The seed preparation protocol was identical for S-AgNWs and L-AgNWs; the only variable was the *M*_w_ of PVP, which governs facet stabilisation and, consequently, the final wire dimensions.

Lower *M*_w_ PVP was found to have faster dissolution rates in EG compared with higher *M*_w_ PVP, primarily due to its lower viscosity and increased chain mobility. However, the stirring RPM was exactly the same for both types of wires. Seed purity and dissolution kinetics showed the same trend for both AgNWs. Similarly, they play decisive roles in determining the nucleation uniformity, growth selectivity, and spectral homogeneity of AgNWs.

Like S-AgNWs, L-AgNWs were initially synthesised using stored AgCl. This resulted in a highly impure product and broadened FWHM (213.6 nm) (Fig. 4A), indicating a wider size distribution and increased by-product formation. Subsequently, when freshly prepared AgCl was employed, a subsequent reduction in FWHM (169.2 nm) was observed (Fig. 4A).

Furthermore, when AgCl is used after adequate grinding for uniform dispersion, the well dispersed, non-aggregated AgCl particles exhibited better dissolution kinetics in EG. In addition, strict regulation of reaction temperature (160 °C) and maintenance of an inert N_2_ atmosphere minimised oxidative side reactions and suppressed heterogeneous nucleation. This caused homogeneous nucleation and uniform NWs growth, as reflected by the shortening of the FWHM. As a result the FWHM reduced from 169.2 to 143.4 nm in the absorption spectrum (Fig. 4C).

In accordance with our working hypothesis, two additional parameters were systematically investigated to promote further anisotropic elongation and reduce polydispersity in L-AgNWs. First, the reaction medium was diluted by increasing the volume of EG relative to that used for S-AgNWs, thereby alleviating spatial confinement and ensuring sufficient free volume for one-dimensional growth. Second, the growth time was extended to provide adequate temporal window for anisotropic stretching of the nanowires.

To optimise the growth duration, the reaction was continued for 150 min. Aliquots were withdrawn at 60, 90, 120 and 150 min (FWHM = 140.4, 137.5, 122.7, and 118.5, respectively), and their UV-vis absorption spectra were recorded (Fig. 4B). At 60 min, the spectrum displayed a sharp peak centred at ∼398 nm with a shoulder at ∼350 nm. These features correspond to the transverse plasmon modes of AgNWs, indicating that anisotropic growth had already commenced. At 90 min, the ∼398 nm peak exhibited a slight red shift (∼3 nm), accompanied by a sharpening of the ∼350 nm shoulder. The red shift suggests an increase in the nanowire length and aspect ratio, while peak sharpening indicates improved morphological uniformity.

Between 120 and 150 min, only a marginal change of 4.2 nm was observed, from 122.7 to 118.5 nm, in the FWHM, implying that most of the NW growth was completed within 120 min. Nevertheless, extending the reaction time to 150 min ensured complete growth and minimised residual nanoparticles and by-products. Notably, the progressive elongation was accompanied by a continuous narrowing of the FWHM from 137.5 to 118.5 nm (Fig. 4B), reflecting reduced polydispersity and improved structural homogeneity compared with previous conditions.

To further decrease the FWHM, all previously optimised parameters were kept constant while refining the thermal and addition protocols. In the abovementioned experiments, the PVP-EG mixture was preheated to 160 °C without strictly controlling the preheating duration. In the refined protocol, the preheating time was standardized to 30 min after the mixture reached 160 °C to ensure complete thermal equilibration and eliminate fluctuations before the addition of the reactants. This modification improved the reproducibility and consistency of the synthesis process. AgCl was introduced in a single portion to promote rapid and homogeneous dissolution during the early reaction stage. The reactor was wrapped with an aluminium foil to minimise thermal fluctuations, and a needle outlet with an N_2_ inlet was employed to release the excess nitrogen oxides formed during the reaction, thereby reducing oxidative side reactions and preserving the product integrity. By incorporating these refinements, seven batches were synthesised to evaluate reproducibility. While reproducing the optimised batch, the FWHM value decreased from 107.3 to 91.2 nm (Fig. 4D). The 8.8 g scale is tested for 4 batches (FWHM = 91.2, 91.6, 97.4, and 101.6 nm; mean = 95.4 ± 5.2), and the 13.2 g scale is tested for 3 batches (FWHM = 102.9, 107.3 and 107.0 nm; mean = 105.3 ± 2.2 nm). This significant narrowing indicates the enhanced monodispersity and highly uniform morphology of the NWs. Importantly, the optimised product exhibited negligible by-product formation, with no post-synthesis purification required for the product. The NWs were directly precipitated using acetone, with only residual polymer removal required.

**Fig. 5:**
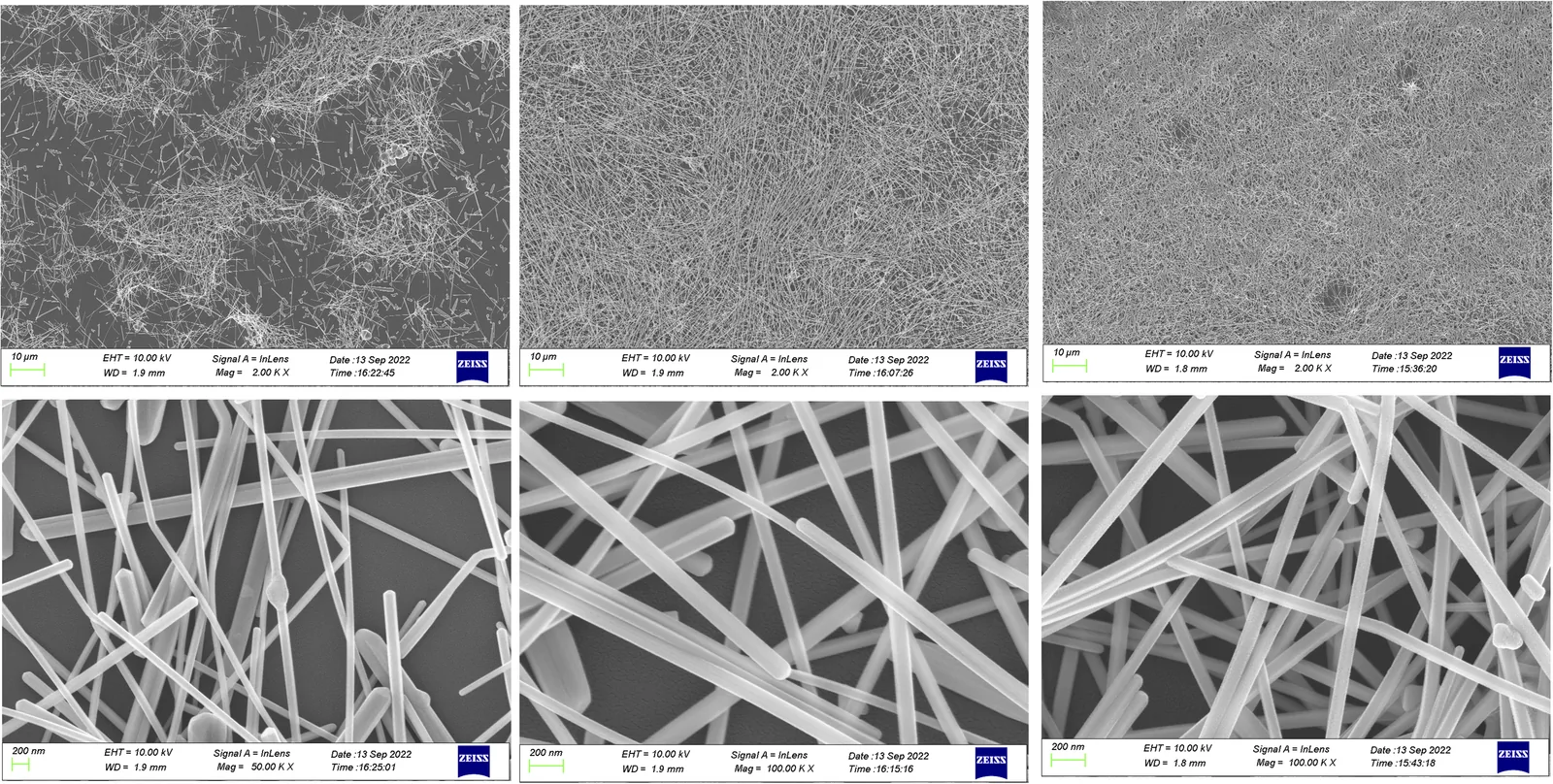
SEM images of the purified L-AgNWs of different batches (purified nanowire diluted in ethanol) at different resolutions: (A and D) L-AgNWs with by-products (broad FWHM = 168 nm, refer to the absorption profile in Fig. 4C), (B and E) optimization batches of L-AgNWs (FWHM = 143.4 nm, refer to the absorption profile in Fig. 4C) and (C and F) the best optimized batch of L-AgNWs (FWHM = 91.2 nm, refer to the absorption profile in Fig. 4D).

To establish a clear correlation between morphology and the FWHM of the absorption profile of L-AgNWs, three independent batches exhibiting distinct absorption profiles were systematically analysed by UV-vis spectroscopy and SEM (Fig. 1b and 5A–F, respectively). The first batch exhibited the broadest FWHM (169.2 nm). As anticipated, SEM analysis revealed significant polydispersity and a high concentration of by-products. The nanowire diameters varied widely from 60–200 nm, while their lengths ranged between 10–30 µm. In addition to the NWs, multiple secondary morphologies were observed, including nanorods, elongated plates, and both thin and thick wires. The broad plasmon band is therefore attributed to the heterogeneous size distribution and mixed morphologies (Fig. 5A and D), which lead to plasmon damping and spectral broadening.

The second batch showed an intermediate FWHM (143.4 nm). Morphologically, this sample exhibited improved uniformity with good precision and reduced by-product formation (Fig. 5B and E). Minor populations of small plates and rods were still present. The NW diameters ranged from 80–200 nm, with their lengths ranged between 20–30 µm. The comparatively narrower FWHM indicates improved growth and reduced size distribution relative to the initial batches.

As we move towards a shorter FWHM, the thickness homogeneity in the bulk morphology improves. For example, the batch with the FWHM of 143.4 nm (Fig. S4) among the optimization batches has a thickness range of 70–115 nm.

The best optimized batch displayed the narrowest FWHM of 91.2 nm (Fig. 1b). SEM analysis confirmed the highly uniform nanowires with minimal secondary structures (Fig. 5C and F). The majority of NWs exhibited diameters of 80 ± 10 nm and lengths were broadly distributed between 20–42 µm (Fig. S1). The isolated wires from the crude reaction mixture have a significantly reduced FWHM, which reflects enhanced morphological uniformity, minimized polydispersity, and suppressed by-product formation.

**Fig. 6:**
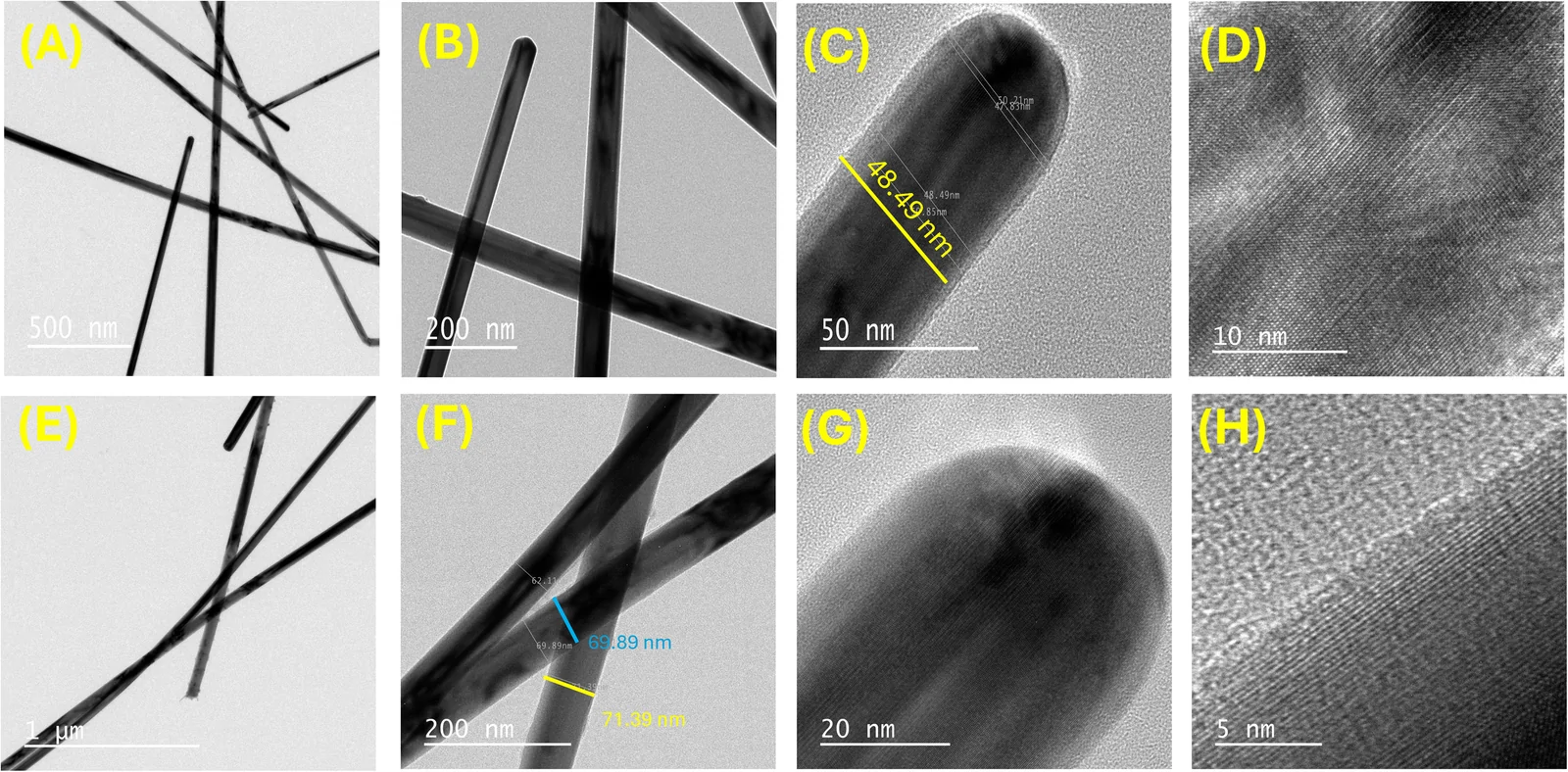
TEM images of the purified thin (A–D) (thickness ≈ 50 nm) and thick (E–H) (thickness ≈ 70 nm) AgNWs at different resolutions.

To further confirm the purity and composition of the synthesized AgNWs, additional characterization, including HRTEM, XRD and TGA analysis, was performed. The morphology and microstructure of the synthesized AgNW batch with the best FWHM (91.2 nm) were investigated by HRTEM, as shown in Fig. 6A–H. The TEM images (Fig. 6(A, B, E and F)) reveal the successful synthesis of two distinct populations of S-AgNWs and L-AgNWs consisting of thin and thick nanowires with smooth, continuous morphologies. S-AgNWs exhibit a uniform diameter of approximately 50 ± 2 nm (Fig. 6B and C), while the L-AgNWs possess an average diameter of 70 ± 4 nm (Fig. 6F and G). No significant presence of silver nanoparticles or other secondary morphologies was observed, confirming the high selectivity of the synthesis. The TEM images (Fig. 6C and G) further demonstrate well-defined rounded end caps, characteristic of AgNWs grown through the polyol-mediated penta-twinned crystal growth mechanism. A well-resolved lattice, extending continuously along the nanowire axis, indicates excellent crystallinity with minimal structural defects (Fig. 6D and H). The absence of grain boundaries or lattice discontinuities suggests that both S-AgNWs and L-AgNWs are predominantly single crystalline. A thin amorphous surface layer surrounding the NWs is attributed to the residual PVP capping agent, which stabilizes AgNWs during growth. Overall, the TEM observations confirm that the modified synthesis protocol produces highly uniform nanowires, with controlled diameters while preserving excellent crystallinity, making them suitable for multigram scale synthesis.

**Fig. 7:**
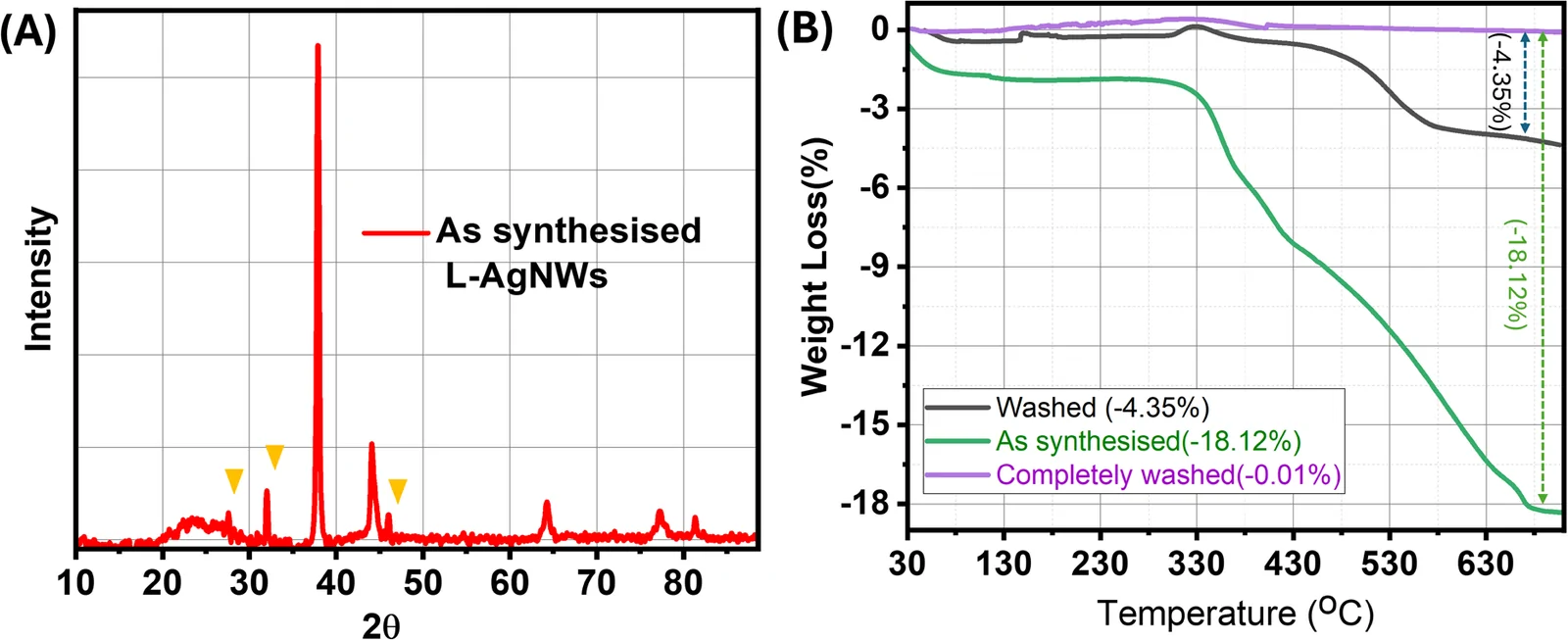
(A) XRD pattern of the as-synthesized AgNWs. The broad hump in the XRD pattern is due to the amorphous PVP capped onto the Ag nanowires. Triangular yellow symbols show the presence of silver chloride used during the nucleation process. (B) TGA curves of the as-synthesized PVP-coated AgNWs (green), partially washed (black) completely washed AgNWs (lilac).

The XRD pattern of the as-synthesized AgNWs confirms the formation of face-centred cubic metallic silver. The diffraction peaks observed at approximately 38.1°, 44.3°, 64.5°, and 77.5° correspond to the (111), (200), (220), and (311) planes of Ag, respectively. The dominant (111) reflection indicates preferential crystallographic growth along the nanowire axis. No distinct diffraction peaks corresponding to Ag_2_O were detected, indicating negligible oxidation during synthesis and purification. Weak reflections near 27.8° and 46.2° can be assigned to trace amounts of AgCl generated during the nucleation process; however, the amount is below the quantitative detection capability of conventional XRD. The broad hump observed in the low-angle region originates from the amorphous PVP coating (Fig. 7A).

Quantitative TGA analysis was also performed. The residual PVP content was estimated to be approximately 18 wt% for crude AgNWs, 4 wt% for optimized AgNWs, and 0.01 wt% after extensive purification (Fig. 7B). Collectively, these results demonstrate that systematic control over dilution, growth duration, thermal history, and reagent addition plays a decisive role in regulating anisotropic growth. Fine-tuning these parameters suppresses competing nucleation pathways, minimizes plasmon damping arising from morphological heterogeneity, and yields highly uniform L-AgNWs suitable for reproducible and scalable synthesis.

### 3.2 Preparation of the transparent conductive film

The best approach to validate the quality of materials is through functionality testing. AgNWs are used to make conductive inks for optoelectronic applications, particularly transparent conductive films (TCFs). Conductive inks for printed electronics are typically composed of three components: a conductive component, such as AgNWs, an organic binder, and a solvent. Alcohol-based printable AgNW inks have gained interest due to their eco-friendly nature.^38,39^ EC is a particularly suitable binder for flexible electronics, exhibiting unique characteristics such as low air permeability and high transparency on polymer substrates like PET. The preparation of the AgNW ink involved adding an ethanolic solution of AgNWs to EC, followed by 40 min of stirring. The ink was coated onto a PET substrate (125 µm thick, 10 × 10 cm, and pre-cleaned) using a wire bar and dried in a hot air oven at 100 °C for 10 min.

**Fig. 8:**
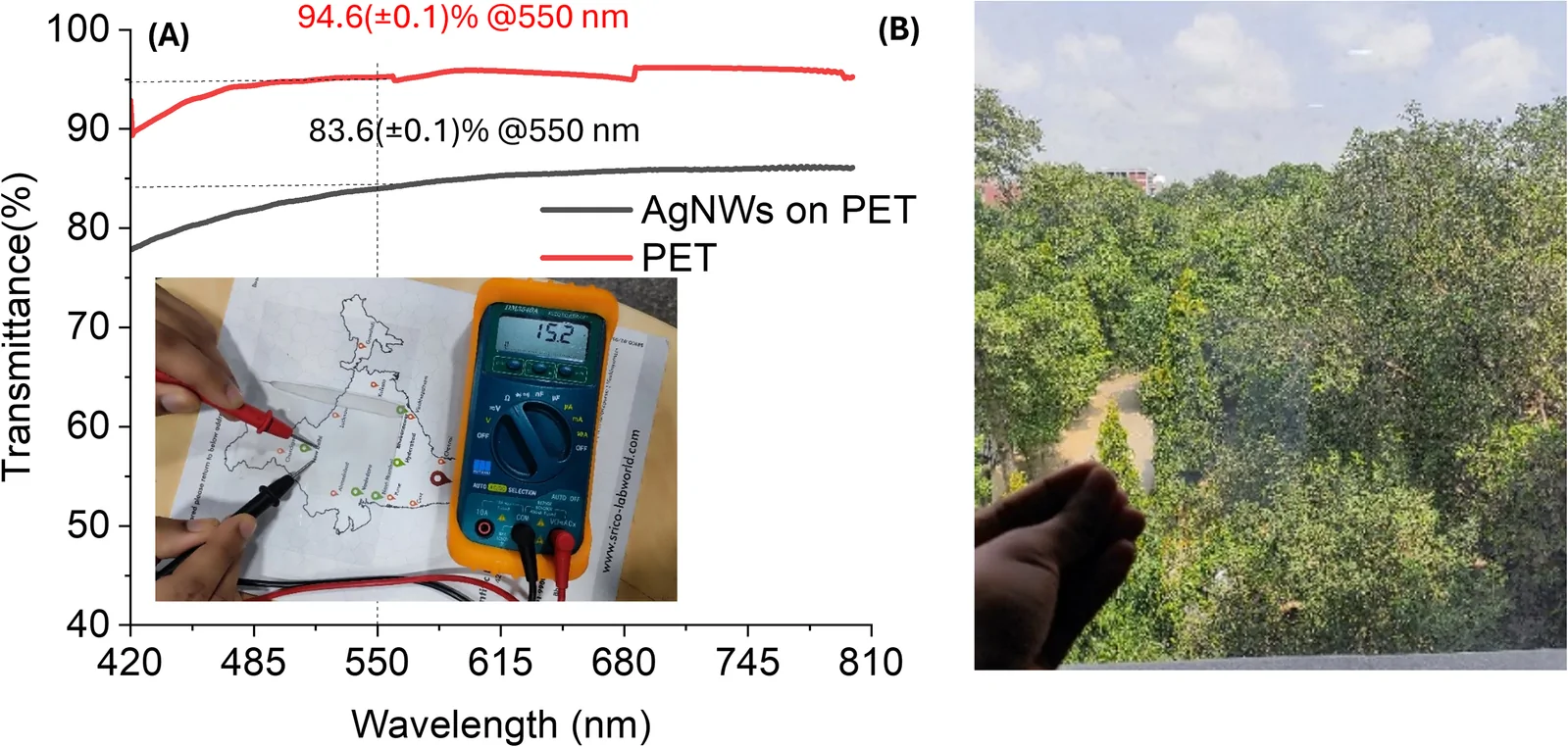
(A) Transmittance of the PET- and AgNW-coated PET films (inset: photograph of the 2 probes). (B) Transparent film of AgNWs coated on 125 µm of a 10 × 10 cm PET substrate by wire bar coating. The photograph was taken by keeping the film on a glass window. The transparency of the film was found to be 88.4% (w.r.t PET).

A preliminary electrical test was conducted to evaluate the functionality of the AgNW film, particularly the sheet resistance. Sheet resistance was measured using the four-probe method. The average sheet resistance was found to be 16.23 Ω sq^−1^. The transmittance reported with reference to air was 83.6% (Fig. 8A). However, the used PET was not fully transparent (*T*% = 94.6%). Accordingly, the exact transmittance was calculated as 83.6/94.6 × 100 = 88.4%.

## 4 Conclusions

This study demonstrates that the reaction conditions optimised at the milligram scale are not directly transferable to the gram-scale synthesis as precise control over the nucleation and growth kinetics is essential for the desired NW morphology and product yield. Increasing the reaction volume introduces additional kinetic variables that significantly influence the NW diameter distribution, aspect ratio, and by-product formation. By systematically addressing these parameters, we established a robust strategy that preserves the uniformity and reproducibility of NWs during scale-up, as confirmed by SEM and UV-vis analyses. The method was optimised for 0.11 g precursor loading per batch to 13.2 g precursor loading per batch (≈120-fold scale-up) without compromising morphology or yield. Yields of up to 91.0% ± 0.8% were observed, demonstrating excellent reproducibility and process reliability. AgNWs were characterised by UV-vis absorption spectroscopy, SEM, TEM, PXRD and TGA. Two well-defined AgNW morphologies were obtained: S-AgNWs (10–15 µm length and 45 ± 10 nm diameter) and L-AgNWs (20–42 µm length and 80 ± 10 nm diameter). To demonstrate their practical applicability, the synthesised AgNWs were formulated into conductive inks compatible with non-porous PET substrates. Uniform wire-bar-coated transparent conductive films exhibited an optical transmittance of about 88.4% w.r.t PET and an average sheet resistance of 16.23 Ω sq^−1^, confirming their suitability for their applications in solution-processed transparent electrodes and flexible electronic devices.

Overall, the present work establishes a simplified and economically viable route for the scalable production of high-quality AgNWs by controlling the kinetic parameters, thereby effectively bridging the gap between laboratory-scale optimisation and gram-scale synthesis. The integrated optimisation of synthesis and downstream processing provides a practical framework for the large-scale production of AgNW-based transparent conductive materials for next-generation flexible electronics.

## Author contributions

Ashish Gupta: funding acquisition, conceptualization and administration of the project, advisor, writing – review, editing and validation. Umesh P Pandey: conceptualization, methodology, experiments, characterization, writing – original draft, review and editing. Y. N. Mohapatra: funding acquisition, writing – review and editing.

## Conflicts of interest

The authors declare no conflicts of interest.

## Supplementary Material

RA-016-D6RA03602C-s001

## Data Availability

The authors confirm that the data supporting the findings of this study are available within the article and its supplementary information (SI). Supplementary information is available. See DOI: https://doi.org/10.1039/d6ra03602c.
