# Supplementary material for: Scalable one-pot synthesis of high-purity silver nanowires via controlled reaction kinetics for transparent conductive ink applications
Source: RSC Adv. 2026 Aug 19;16(41):48983–95. doi: 10.1039/d6ra03602c (PMC13487952; doi:10.1039/d6ra03602c)
Supplement: Supplementary file 1 [file RA-016-D6RA03602C-s001.pdf]

Supplementary Information (SI) for RSC Advances.
This journal is © The Royal Society of Chemistry 2026

SUPPORTING INFORMATION

Scalable one-pot synthesis of high purity silver nanowires via controlled reaction kinetics for transparent conductive ink applications

Umesh Pratap Pandey, Yashowanta N. Mohapatra and Ashish Gupta*

Table of Contents

S1: Step-by-step protocol for AgNWs synthesis

S1A: Synthesis of AgCl

S1B: Synthesis of AgNWs

S1C: Post synthesis work-up

S1D: Storage condition

Figure S1 SEM image of crude nanowire to show the length(above). A zoomed image of a wire having a length 42.28 micrometers (below.)

Figure S2 A zoomed image of a wire **Figure S1** having a length 42.28 micrometers (below.)

Figure S3 Effect of Aging on UV-Vis of L-AgNWs over 150 days storage

Figure S4 bulk morphology and thickness of silver nanowire at different resolutions After washing the silver nanowire with ethanol and water (amount of PVP around 4%)

Figure S5: Formation of Nano Aggregates over a year, when kept under ambient condition in Ethanol.

Table S1 Calculation of Resistivity and Conductivity from Sheet Resistance and Calculation Method.

S1: Step-by-step protocol for AgNWs synthesis

S1A: Synthesis of AgCl

Caution! Synthesis must be performed in the dark.

- Make 0.5 M (DD water) aqueous solution of silver nitrate
- Make 1 M (DD water) aqueous solution of common salt.
- Mix common salt solution into silver nitrate under stirring for 1 min, AgCl immediately flocculates.
- The precipitate is separated from the supernatant, wash twice with DD water.
- Material should be dried in the dark.
- Yield should come 100%.

Calculation of concentration:

Weight of solute (grams)= Molarity x Mol wt. of solute x Volume of solvent(ml)/1000

Or

Weight of solute (mg) = Molarity x Mol wt. of solute x Volume of solvent(ml)

Example:

Weight of AgNO3= 0.5 x 169.9x 30ml/1000 =2.548 grams

Weight of NaCl= 1 x 58.5x 30ml/1000 =1.755 grams

Precautions: AVOID sunlight throughout the process.

S1B: Synthesis of AgNWs

- Take Clean and dry reactor equipped with N_2 line and Heater.
- Add Ethylene Glycol and start stirring at RPM =450
- Add PVP slowly with continuous stirring.
- Heat the reaction mixture at160 °C.
- Wait until PVP dissolve completely. It may take 30-40 minutes. Wait till the reaction mixture becomes transparent. (i.e. no lumps of PVP left)
- When there are no fluctuations in temperature, Add AgCl (**Dry, grinded stored in dark)** in one shot with continuous stirring.
- Wait **exactly 3 min** (be careful), add $AgNO_3$ one shot (Not more than 1 minute).
- After adding AgCl, Color changes from transparent to pale yellow which indicate seed formation.
- After adding $AgNO_3$, the color changes from pale yellow to silver grey. And reaction mixture gradually became dense.

- Reflex the reaction mixture (time for particular reaction scale is given in table 1). After completing the reaction turn off the heater
- Let the reaction cool down up to 70-80 °C
- Add 50 ml ethanol slowly while continuously stirring to quench the reaction. **Be Careful Quenching is an exothermic process.**
- Cool down the reaction mixture to RT with continuous stirring.
- Collect the product in a storage bottle.
- Turn off the stirrer.
- Crude Wire is ready, take 30µl and dilute 100 times in ethanol without any purification record UV and compare with standard UV.
- **Quick confirmatory test:** Take 0.5 ml of as synthesized AgNWs in 100 mL breaker. Add 10 ml acetone. If viscus solid precipitate forms, the product could be AgNWs.

S1C: Post synthesis work-up:

Post synthesis work-up of silver nanowires is a crucial step to get the desired functionalities. Here is a general guideline on how one can wash silver nanowires to remove impurities, residues, or excess PVP from the surface of the nanowires:

- Take crude synthesised AgNWs in 1 big beaker. Add acetone slowly until wire start precipitating. Wait until AgNWs get precipitated.
- In general, it requires 1:4 (V/V) AgNWs : acetone, to get the wire precipitated.
- Take out the supernatant acetone and wash the precipitate with acetone twice. (mild white)

Now we have pure AgNWs with lot of PVP (PVP is insoluble in acetone)

- Disperse the precipitate in water, ethanol mixture (1:1), by using magnetic starring.
- Keep stirring disperse until all the precipitate get dispersed in ethanol water uniformly. No residue is left at the bottom of the beaker.
- Left the beaker for 20 minutes to get the heavy particles precipitated. (residue of AgCl and other byproducts)
- Centrifuge the dispersed wire at 3000 rpm for 15mins.
- As per need, Second & third washing may to reduce PVP, up to 1.5 to 2%.

S1D: Storage condition

Silver nanowires with residual PVP coating on the surface of AgNWs can act as a protective layer to prolong the storage time. Disperse PVP coated AgNWs in ethyl alcohol can be stored at 5 °-15° C.

Store in dark and dry places. Absorption profile of large nanowire before and after 150 days of storage to see the effect of Aging of L-AgNWs (Figure: S3)

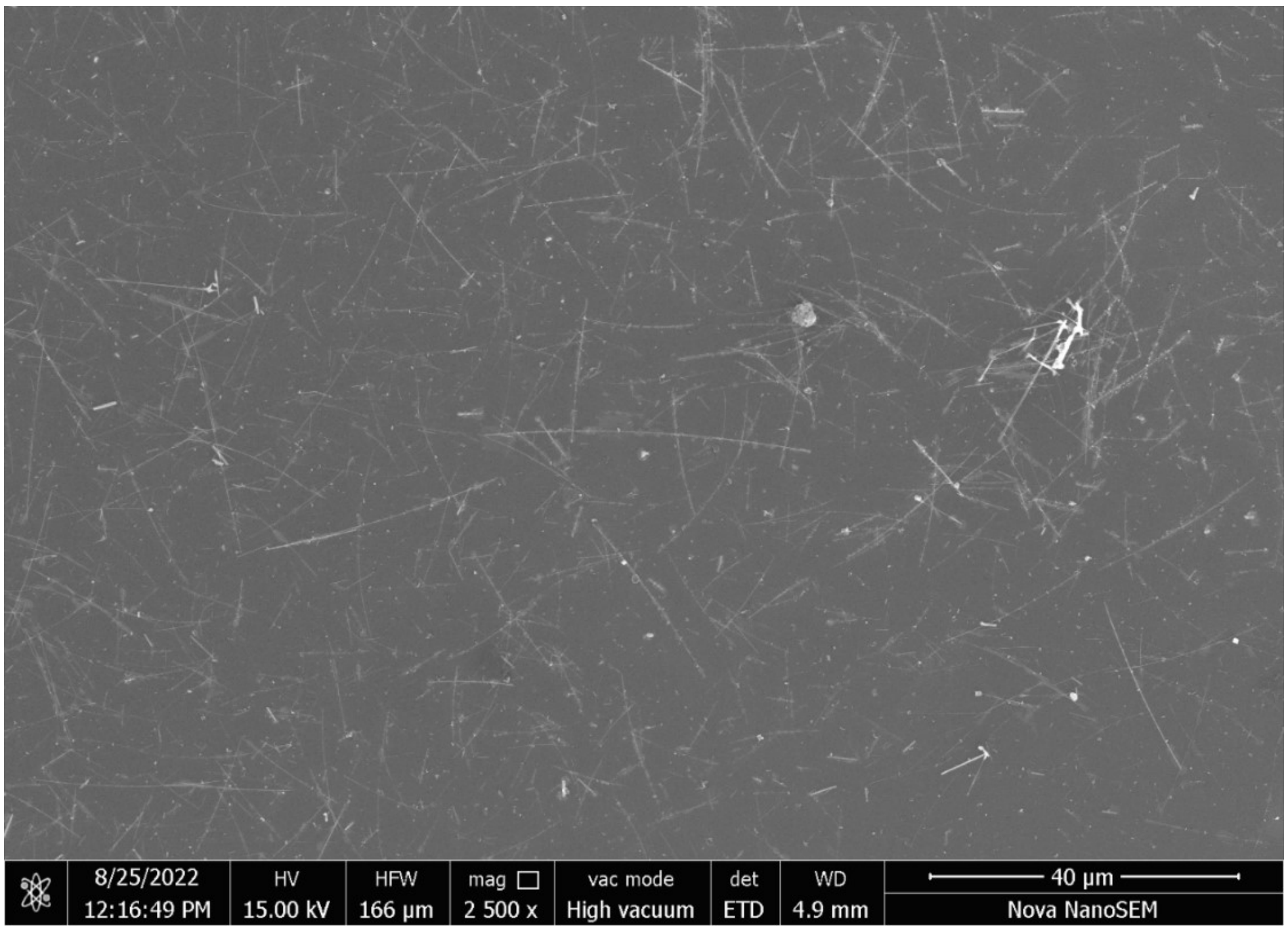

Figure S1 SEM image of crude nanowire to show the length (above). A zoomed image of a wire having a length 42.28 micrometers (below.)

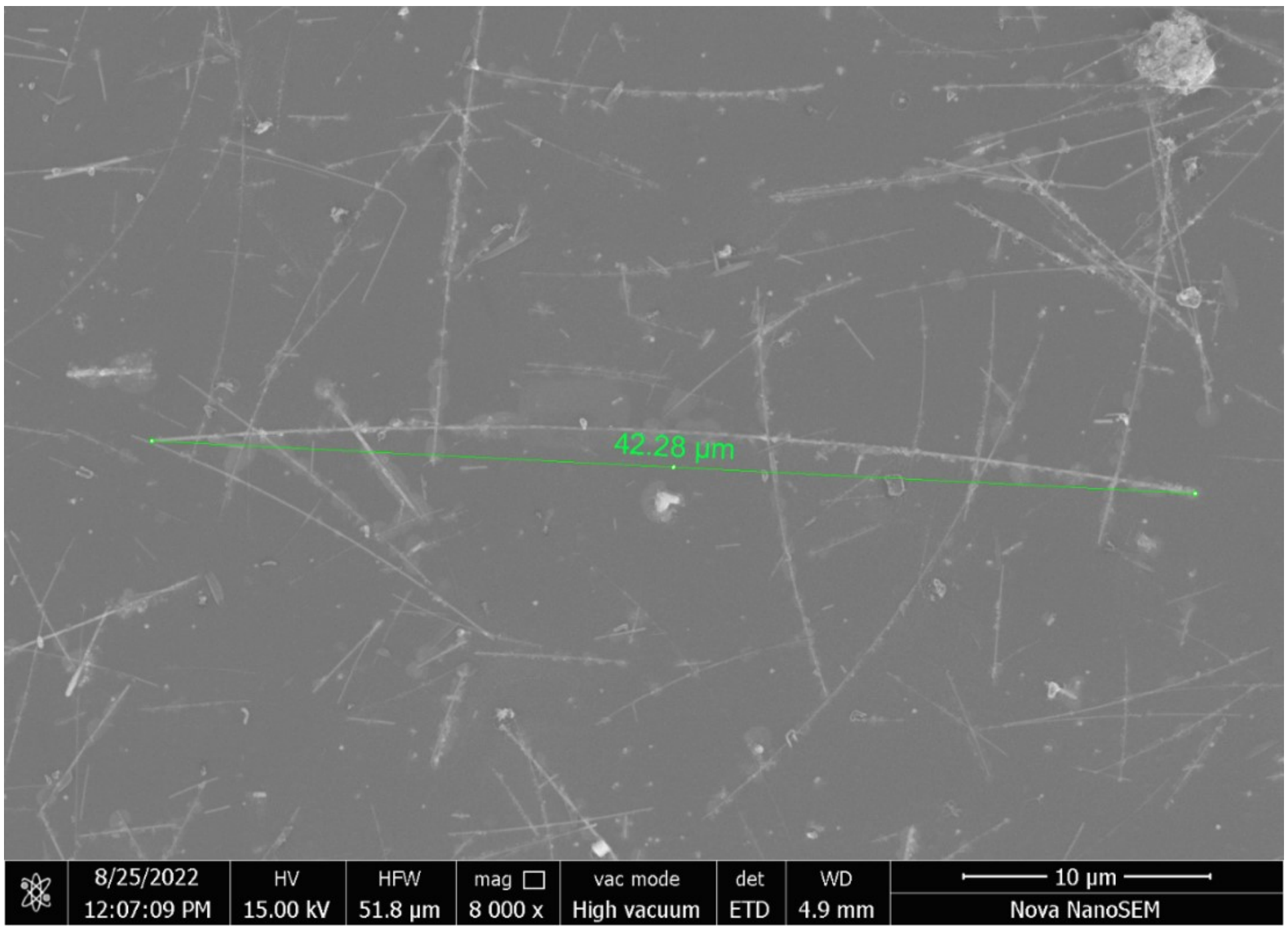

Figure S2 A zoomed image of a wire **Figure S1** having a length 42.28 micrometers (below.)

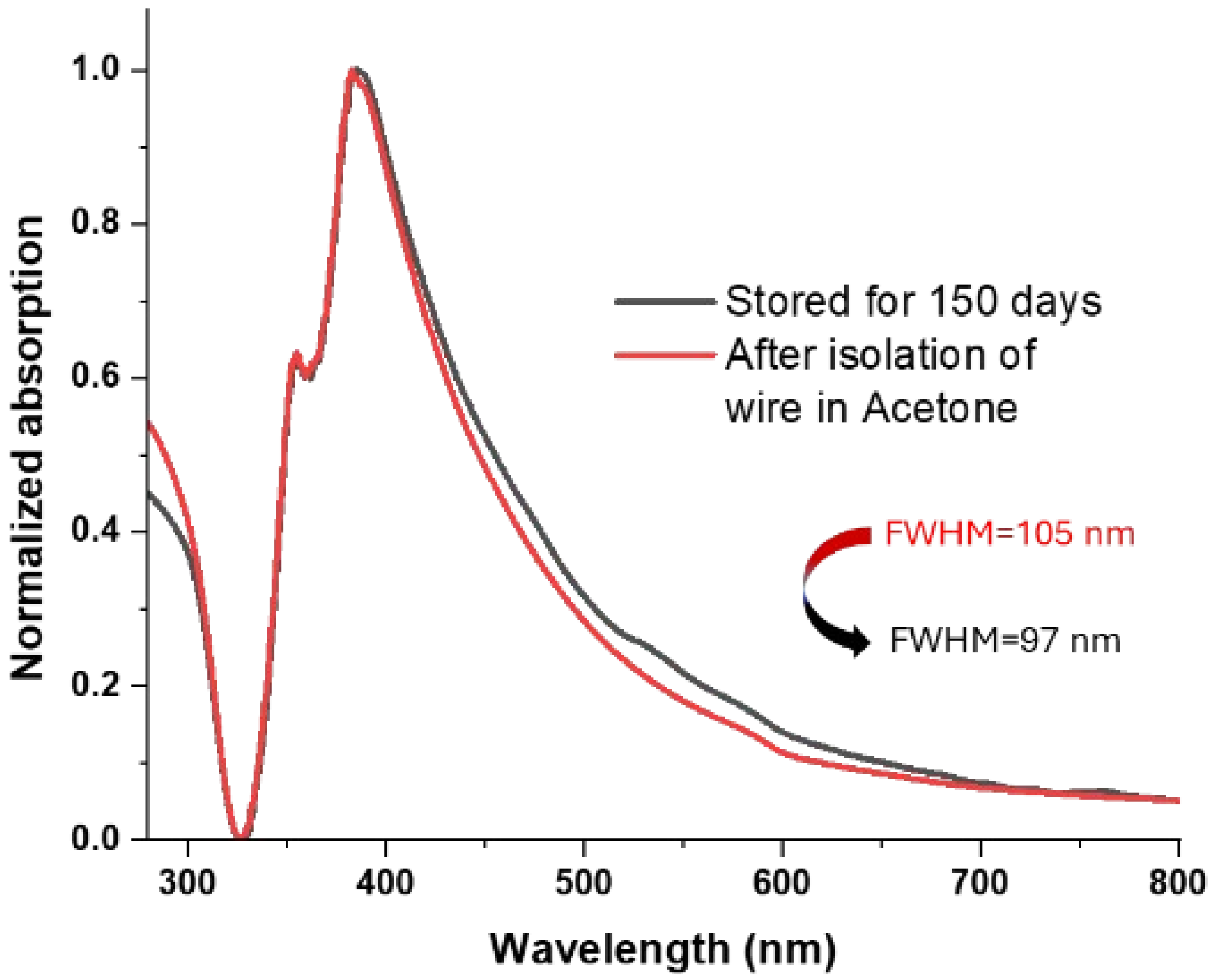

Figure S3 Effect of Aging on UV-Vis of L-AgNWs over 150 days storage

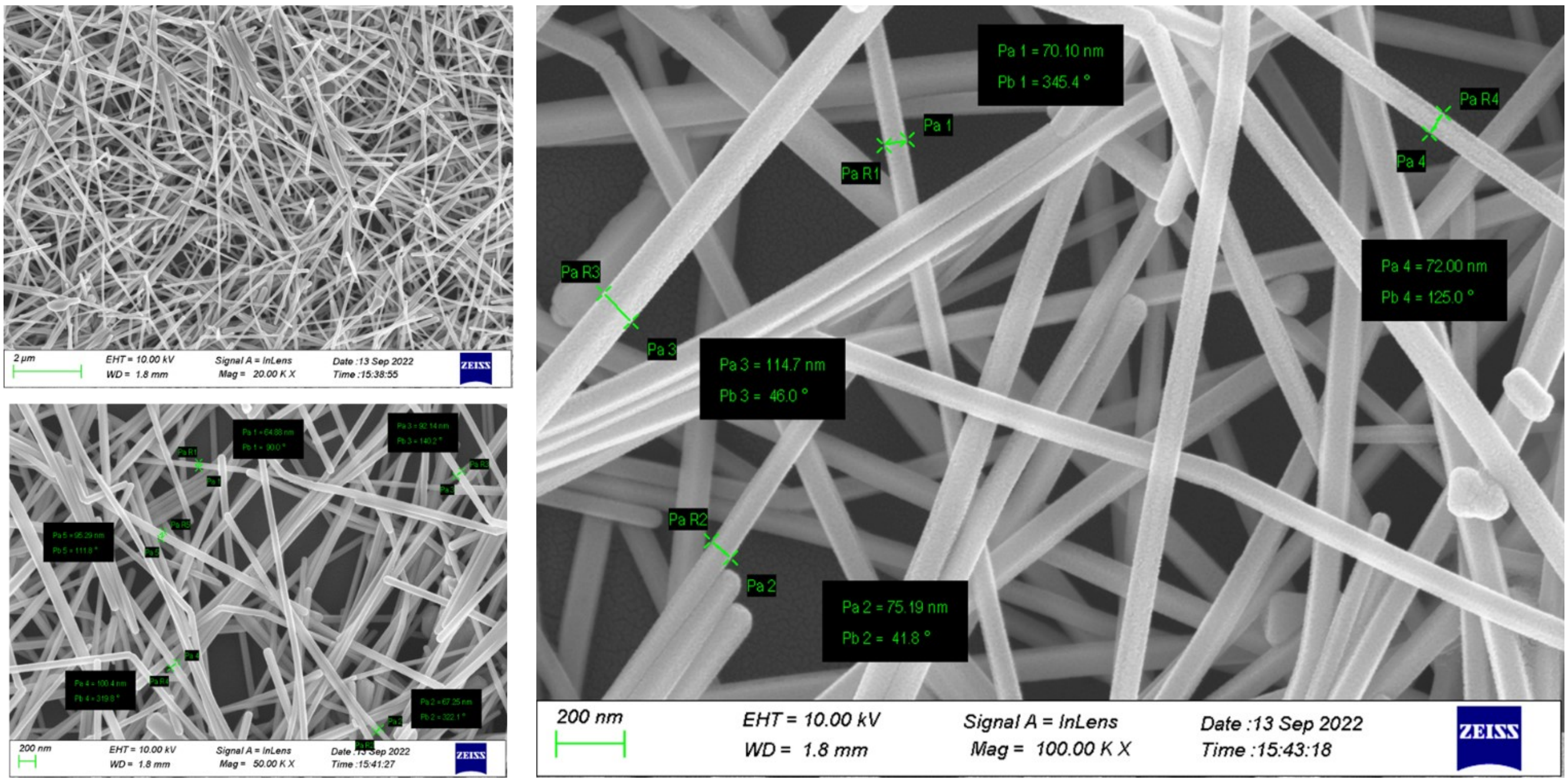

Figure S4 bulk morphology and thickness of long silver nanowire at different resolutions After washing the silver nanowire with ethanol and water (amount of PVP 1to 2%)

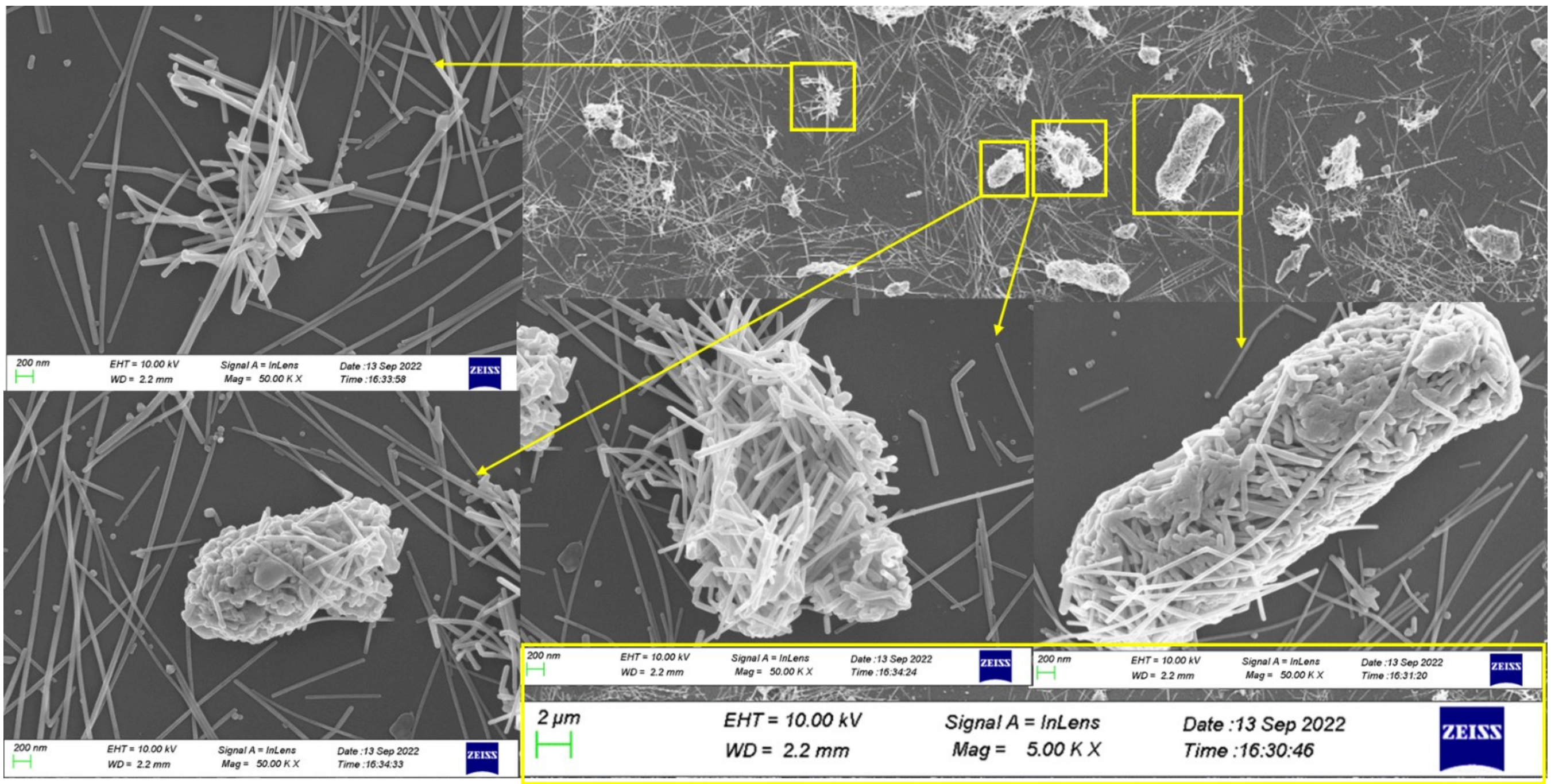

Figure S5: Formation of Nano Aggregates of thin nanowire over a year, when kept under ambient condition in Ethanol.

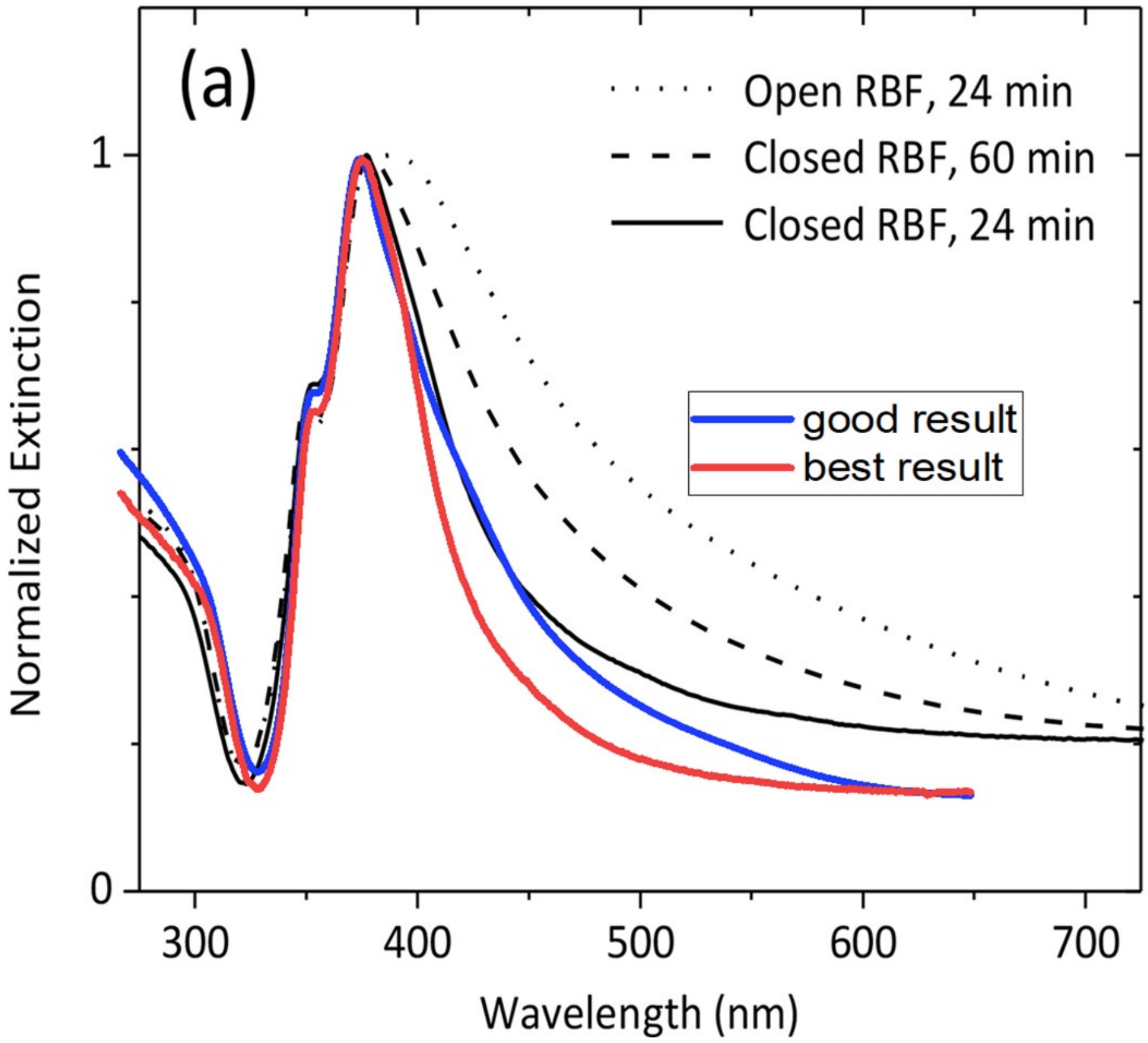

Figure S6: Comparison of normalized excitation AgNWs were compared with best optimized results by with Baldi and coworkers.

Calculation of Resistivity and Conductivity from Sheet Resistance

Calculation Method

1. Sheet resistances were Measured using a four-point probe.
2. Convert film thickness to m.
3. Calculate resistivity using ρ = Sheet resistances (R_s) × thickness (t)
4. Conductivity Calculated using σ = 1/ρ.
5. Reported the average values at the end of the table.

AgNWs film thickness (t) = 200 nm = 200×10^{-9} m = 2.0×10^{-7} m

Formula:

Sheet resistance (R_s) is related to resistivity (ρ) by:
$R_s = \rho / t$

Therefore,
$\rho = R_s \times t$

Electrical conductivity (σ) is the reciprocal of resistivity:
$\sigma = 1/\rho$

Sample Calculation (R_s = 17.03 Ω/sq)

$\rho = 17.03 \times 2.0 \times 10^{-7} = 3.406 \times 10^{-6}$ Ω·m

$\sigma = 1/(3.406 \times 10^{-6}) = 2.94 \times 10^{5}$ $S \cdot m^{-1}$

Calculated Results

Sheet Resistance (Ω/□)	Resistivity (Ω·m)	Conductivity ($S \cdot m^{-1}$)
8.40	1.68 $X10^{-6}$	5.95 $X10^{5}$
11.90	2.38 $X10^{-6}$	4.20 $X10^{5}$
17.03	3.41 $X10^{-6}$	2.94 $X10^{5}$
17.54	3.51 $X10^{-6}$	2.85 $X10^{5}$
26.28	5.26 $X10^{-6}$	1.90$X10^{5}$
**Average = 16.23**	**Average = 3.25 $X10^{-6}$**	**Average = 3.57 $X10^{5}$**

Table: S1 Four probe data of ink functionality testing.

Note:
The deposited films showed thickness 200 nm with variation of approximately 50 nm (200±50 nm) due to wire bar coating. The ink was coated onto a PET substrate (125µm thick, 10x10cm pre-cleaned) using wire bar and dried in hot air oven at 100oC for 10min.
